# A Comprehensive Analysis of Fibrillar Collagens in Lamprey Suggests a Conserved Role in Vertebrate Musculoskeletal Evolution

**DOI:** 10.3389/fcell.2022.809979

**Published:** 2022-02-15

**Authors:** Zachary D. Root, Cara Allen, Claire Gould, Margaux Brewer, David Jandzik, Daniel M. Medeiros

**Affiliations:** ^1^ Department of Ecology and Evolutionary Biology, University of Colorado, Boulder, CO, United States; ^2^ Department of Zoology, Comenius University in Bratislava, Bratislava, Slovakia

**Keywords:** lamprey, fibrillar collagen, evolution, cartilage, development

## Abstract

Vertebrates have distinct tissues which are not present in invertebrate chordates nor other metazoans. The rise of these tissues also coincided with at least one round of whole-genome duplication as well as a suite of lineage-specific segmental duplications. Understanding whether novel genes lead to the origin and diversification of novel cell types, therefore, is of great importance in vertebrate evolution. Here we were particularly interested in the evolution of the vertebrate musculoskeletal system, the muscles and connective tissues that support a diversity of body plans. A major component of the musculoskeletal extracellular matrix (ECM) is fibrillar collagens, a gene family which has been greatly expanded upon in vertebrates. We thus asked whether the repertoire of fibrillar collagens in vertebrates reflects differences in the musculoskeletal system. To test this, we explored the diversity of fibrillar collagens in lamprey, a jawless vertebrate which diverged from jawed vertebrates (gnathostomes) more than five hundred million years ago and has undergone its own gene duplications. Some of the principal components of vertebrate hyaline cartilage are the fibrillar collagens type II and XI, but their presence in cartilage development across all vertebrate taxa has been disputed. We particularly emphasized the characterization of genes in the lamprey hyaline cartilage, testing if its collagen repertoire was similar to that in gnathostomes. Overall, we discovered thirteen fibrillar collagens from all known gene subfamilies in lamprey and were able to identify several lineage-specific duplications. We found that, while the collagen loci have undergone rearrangement, the Clade A genes have remained linked with the *hox* clusters, a phenomenon also seen in gnathostomes. While the lamprey muscular tissue was largely similar to that seen in gnathostomes, we saw considerable differences in the larval lamprey skeletal tissue, with distinct collagen combinations pertaining to different cartilage types. Our gene expression analyses were unable to identify type II collagen in the sea lamprey hyaline cartilage nor any other fibrillar collagen during chondrogenesis at the stages observed, meaning that sea lamprey likely no longer require these genes during early cartilage development. Our findings suggest that fibrillar collagens were multifunctional across the musculoskeletal system in the last common ancestor of vertebrates and have been largely conserved, but these genes alone cannot explain the origin of novel cell types.

## Introduction

The musculoskeletal system is a complex network of tissues which support the body and allow a broad range of motions. Composed of muscles and various connective tissues like skeleton, tendons, and ligaments, it has diversified across metazoans, permitting new morphologies and body plans. The vertebrate lineage has expanded considerably upon this musculoskeletal system, incorporating cytoskeletal and extracellular matrix (ECM) proteins which have diversified these tissues and their mechanical properties. These populations of musculoskeletal tissue have distinct functions that influence the vertebrate trunk, viscera, and head in different ways (Nathan et al., 2008; Harel et al., 2009; Sambasivan et al., 2009; Witten et al., 2010). Understanding how these tissues have diversified from those seen in invertebrate chordates has significant value in vertebrate evolution.

Vertebrates are thought to have undergone at least one whole-genome duplication, and gene duplication is an important mechanism through which they may have elaborated this musculoskeletal morphology (Van de Peer et al., 2009; Ohno, 2013; Martik et al., 2019; Simakov et al., 2020; Square et al., 2020). Although the metazoan muscular system’s origins are thought to be shared and innovated upon an ancestral contractile machinery (Seipel and Schmid, 2005; Steinmetz et al., 2012), vertebrates have duplicated actin and myosin heavy chain genes which are differentially expressed across smooth and striated muscle populations (Vandekerckhove and Weber, 1984; Mugue and Ozernyuk, 2006; Warkman et al., 2012; Witjes et al., 2019), forming the vertebrate-unique cardiac muscle and multi-layered skeletal muscles (Zoghbi et al., 2008; Shaffer and Gillis, 2010; Luther and Squire, 2014). Likewise, cartilage may be a connective tissue shared by bilaterians (Tarazona et al., 2016), but vertebrates have formed unique ECM-rich hyaline cartilages characterized by lectican proteins, all of which are likely vertebrate-specific duplications (Root et al., 2021a). Although tendons connect muscle and skeleton in both invertebrates and vertebrates, they are structurally different in both groups and have different developmental origins (Schweitzer et al., 2010), vertebrate tendons are often heterogeneous in morphology, but share their expression of the vertebrate-specific small interstitial leucine-rich repeat proteoglycans (SLRP’s) like fibromodulin, biglycan, decorin, and lumican (Yoon and Halper, 2005). All of these genes have been found across vertebrates but not invertebrate chordates like amphioxus, supporting the idea that these tendon-associated gene families arose from vertebrate-specific duplications (Matsushima et al., 2000). While aspects of vertebrate evolution are thought to have been predicated on gene duplication, it is difficult to find a single gene family that vertebrates have duplicated which specifically encompasses the musculoskeletal system and its various components.

It is because of this disparity that we were interested in investigating fibrillar collagens. They are the most abundant proteins in vertebrate bodies and are found all across the vertebrate musculoskeletal system (Bailey, 1968). These collagens confer biomechanical properties like load bearing, tensile strength, and torsional stiffness, and these proteins can even act as ligands or receptors for various signaling pathways (Di Lullo et al., 2002; Boraschi-Diaz et al., 2017; Huang et al., 2019). While fibrillar collagens have been found across all metazoans, this gene family has undergone multiple rounds of duplication in vertebrates (Exposito et al., 2010). Modern jawed vertebrates have eleven or twelve fibrillar collagen genes normally, and these are divided into three clades: Clade A (*col1a1*, *col1a2 col2a1*, *col3a1* and *col5a2*), Clade B (*col5a1*, *col5a3*, *col11a1*, and *col11a2*), and Clade C (*col24a1* and *col27a1*). These collagens are dispersed throughout the musculoskeletal system, and are major components of connective tissues. In gnathostomes, *col2a1* is the predominant collagen in cartilage and *col1a1* in bone, but the other genes have important if understudied roles. Furthermore, *col2a1* and *col1a1* have more pleiotropic expression than cartilage and bone exclusively. Previous work has identified *col1a1* in developing tendons, dermis, and perichondrium (Fisher et al., 2003; Grimaldi et al., 2004; Leéjard et al., 2007; Enault et al., 2015; Gistelinck et al., 2016) while *col2a1* has been reported in the perichondrium, notochord, and even in teleost osteocytes, although this bone expression is considerably lower than in cartilage (Eames et al., 2012; Enault et al., 2015). *col1a2* is an alternate alpha chain of type I collagen that shares similar expression to its counterpart in bone, perichondrium, dermis, and tendons, and is also found in developing cartilage and skeletal muscle (Le Guellec et al., 2003; Shukunami et al., 2008; Gaspera et al., 2009; Eames et al., 2012; Gelse et al., 2012; Chen and Galloway, 2014; Enault et al., 2015; Sharma et al., 2019; Fan et al., 2020). *col3a1* is found primarily in developing dermis and tendons, but is also expressed in skeletal muscle, bone, and cartilage, albeit in much lower quantities (Leéjard et al., 2007; Duance et al., 1977; Kuo et al., 2008; D’hondt et al., 2018; Owens et al., 2016; Session et al., 2016; Guerquin et al., 2013; Liu et al., 1997; Wang et al., 2020). Lastly, *col5a2* is a minor component in almost all the aforementioned tissues, with lower levels of expression in cartilage, dermis, bone, and muscle (Rauch et al., 2003; Thisse, 2005; Session et al., 2016). Clade B genes share similar patterns of expression to their Clade A counterparts. *col11a1* is a complementary collagen to Type II in gnathostome cartilage (Li et al., 1995; Yoshioka et al., 1995; Baas et al., 2009), but is also localized in tendons, bone, perichondrium, notochord, and dermis (Yoshioka et al., 1995; Imamura et al., 2000; Thisse, 2005; Baas et al., 2009; Fang et al., 2010; Mukhi et al., 2010; Wenstrup et al., 2011; Suzuki et al., 2012; Sun et al., 2020). *col5a1* is another major Clade B collagen, with expression in the aforementioned tissues as well as developing muscle (Lincoln et al., 2006; Roulet et al., 2007; Bandyopadhyay et al., 2008; Gaspera et al., 2009; Fang et al., 2010; Hoffman et al., 2010; Wenstrup et al., 2011; Session et al., 2016; Sharma et al., 2019). *col11a2* and *col5a3* are poorly documented genes, as one or the other is often absent from gnathostome genomes. Their patterns of expression, however, closely resemble other Clade B genes, with *col11a2* identified in developing cartilage, bone, perichondrium, notochord, and tendons (Tsumaki and Kimura, 1995; Iwai et al., 2008; Fang et al., 2010; Eames et al., 2012; Duran et al., 2015; Cao et al., 2018; Lawrence et al., 2018; Sun et al., 2020) while *col5a3* is expressed in the aforementioned tissues as well as the dermis and skeletal muscle (Imamura et al., 2000; Yamaguchi et al., 2005; Fang et al., 2010; Hoffman et al., 2010; Kessels et al., 2014). The Clade C genes are a more recent discovery in this gene family (Koch et al., 2003; Pace et al., 2003), and their expression is poorly understood. What few studies exist, however, have found *col24a1* musculoskeletal expression in the developing bone (Koch et al., 2003; Wang et al., 2012; Duran et al., 2015) whereas *col27a1* was identified in cartilage, notochord, and some populations of muscle (Thisse et al., 2001; Pace et al., 2003; Jenkins et al., 2005; Christiansen et al., 2009; Mangos et al., 2010; Lien et al., 2013; Duran et al., 2015). While most vertebrates have twelve fibrillar collagen genes with diversified expression, only four have been found in invertebrate chordates, with each gene having largely overlapping expression in the oral ciri, notochord, muscles, and the nerve cord (Wada et al., 2006; Zhang and Cohn, 2006; Jandzik et al., 2015). If gene duplication played anportant role in the early evolution of vertebrate morphology, then structural proteins like fibrillar collagens may have co-diversified in the musculoskeletal system during this process.

The evolution of vertebrates from invertebrate chordates coincided with genomic expansion, but we know little as to how these genes acquired new roles or sub-functionalized previous ones. It is commonly accepted that at least one whole-genome duplication happened at the stem of the vertebrate lineage (Panopoulou and Poustka, 2005; Smith and Keinath, 2015; Simakov et al., 2020), but subsequent duplications may have occurred after extant vertebrate taxa diverged, meaning that some fibrillar collagens may be unique homologs with distinct expression domains. It has been recently suggested that jawed vertebrates (gnathostomes) and extant jawless vertebrates like lamprey and hagfish (cyclostomes) diverged before the second round of whole genome duplication (Simakov et al., 2020), meaning that many fibrillar collagens would be specific to each lineage. Gnathostomes and cyclostomes are thought to have diverged more than 500 million years ago (Kuraku and Kuratani, 2006), providing us an excellent opportunity to explore lineage-specific duplication and functionalization of collagens at one of the earliest nodes in vertebrate evolution. Both lamprey and hagfish lack epaxial-hypaxial divisions in somitic skeletal muscle like that seen in gnathostomes (Peters and Mackay, 1961; Kusakabe and Kuratani, 2007), and they additionally lack appendicular musculature from the lateral plate mesoderm (Nakao, 1977). Furthermore, cyclostomes have independently duplicated muscle actins and myosin chains which are differentially expressed in skeletal muscle, contrasting the somitic and head musculature from each other (Kusakabe et al., 2004). The head mesoderm of lampreys undergoes a split as its dorsal and ventral parts migrate around the eye as supraoptic and infraoptic streams; it has been previously shown that these muscles differentially express the myogenic markers *pax3-7* and *lbx-A* (Kusakabe and Kuratani, 2005; Kusakabe et al., 2011). The tendons of the myosepta also differ greatly between both groups, as hagfish are thought to lack myoseptal tendons entirely while lamprey have only some of this structure, specifically the dorsal and ventral myorhabdoid tendons (Gemballa and Vogel, 2002). The gnathostome and cyclostome skeleton differ as well in that not only does the latter lacks bone and traditional hypertrophic cartilage, but it is also largely heterogeneous throughout the body and has acquired novel proteins in its extracellular matrix such as lamprin, myxinin, and pharymprin (Wright et al., 1983; Wright et al., 1984; Robson et al., 1993; McBurney et al., 1996; Yokoyama et al., 2019). Cyclostome cartilages are also distinct in that some species may no longer require type II collagen during early development. In the arctic lamprey *Lethenteron camtschaticum*, *col2a1b* transcripts have been identified in pharyngeal cartilage during early chondrogenesis (Ohtani et al., 2008). Conversely, type II collagen was reported in *P. marinus* in one study of the larval and adult hyaline cartilages (Zhang et al., 2006), but additional histological analyses were unable to corroborate any *col2a1a* or *col2a1b* expression during early development (McCauley, 2008; Cattell et al., 2011). Likewise, studies in adult hagfish have not found type II collagen in “hard” cartilage, thought to be homologous to gnathostome hyaline cartilage (Zhang and Cohn, 2006; Ota and Kuratani, 2010). These suggest that the sea lamprey have either deviated considerably from the ancestral vertebrate skeleton or have retained features of its diverse cartilages that were later lost in gnathostomes, a phenomenon previously noted with mineralized tissues in stem vertebrates (Eames et al., 2020). Overall, there are notable differences between the vertebrate and invertebrate chordate musculoskeletal system and even between that of cyclostomes and gnathostomes, and lineage-specific gene duplications may have facilitated an important role in these processes by permitting.

In this study, we used genomic and transcriptomic data from the sea lamprey *Petromyzon marinus* and the inshore hagfish *Eptatretus burgeri* to gain insight into the evolutionary history of fibrillar collagen genes and their activity in the musculoskeletal system, particularly in the branchial hyaline cartilage. We identified several lineage-specific duplications and deletions in cyclostomes, with linkage to other important gene families. We find mosaic expression of these genes across the larval lamprey skeleton, corresponding to previously documented subpopulations of cartilage, but we did not identify type II collagen in the developing pharyngeal chondrocytes. We also find a distribution of collagens across the early muscular system, dispersed throughout the dermomyotome as well as differential expression between the anterior and posterior myomeres.

## Results

### Identification of Fibrillar Collagens in Cyclostomes

We first searched for fibrillar collagens through transcriptomic reads of *P. marinus* and *E. burgeri* and verified their position on distinct genomic scaffolds. We identified 13 fibrillar collagens in lamprey: seven Clade A’s, five Clade B’s, and one Clade C. For hagfish, we identified 13 genes: six Clade A’s, five Clade B’s, and two Clade C’s. Before analyzing orthology, all fibrillar collagens were named according to their clade and the order in which they were identified in our transcriptome (*colA1, colA2, colB1*, etc).

All cyclostome fibrillar collagens display a similar protein architecture to their respective families. Most Clade A genes have Von Willebrand Factor Type C (VWC) domain at their N-terminus, almost all Clade B and C genes have a Thrombospondin-1 domain (TSPN) at their N-terminus, and all genes have the archetypal fibrillar collagen COLF C-terminal domain. The main exceptions to this domain architecture were the hagfish *colC2* gene, which did not have the TSPN domain, and the lamprey *colA4* and *colA7* genes, which do not possess the VWC module. These lamprey features are shared with the gnathostome *col1a2* gene, which has also been reported to not have the VWC domain in humans (Aouacheria et al., 2004). Upon reviewing the domain architecture of multiple *col1a2* orthologs, our findings support this observation across gnathostomes. The lamprey *colA4*, hagfish *colA3*, and hagfish *colA4* genes also lack the minor helix domain in the N-terminus which has been conserved across all other Clade A and Clade B genes but is notably absent from vertebrate Clade C genes. The COLF domain has been highly conserved across gnathostome and contains a recognition sequence with eight interspersed cysteine residues which are thought to mediate collagen trimer formation (Lees et al., 1997), and all lamprey and hagfish genes have maintained six of the eight residues (positions 1,4,5,6,7,8). Residue two is modified in the lamprey *colA2*, *colB4*, and *colB5*, and is changed in the hagfish *colA2* and *colA4* genes. This particular cysteine residue is known to be modified across gnathostome genes, specifically *col1a2* and various Clade B collagens across different species (Lees et al., 1997; Exposito et al., 2010). Residue three is only modified in mammalian *col5a2* orthologs, but this change can be found in the lamprey *colA1*, *colA2*, and *colA4* genes, and is also distinct in the hagfish *colA4* and *colB2* genes. Overall, we find significant differences in the structure of cyclostome fibrillar collagen genes compared to other vertebrates on the amino acid level. How these structural differences influence their function, however, is beyond the scope of this study, but will be of future interest.

### Phylogenetic and Syntenic Analysis of Cyclostome Fibrillar Collagens

We next performed phylogenetic analyses on the fibrillar collagens identified, with separate analyses for each clade. We reasoned that, if orthologs could be determined between lamprey and gnathostome genes, we could posit a better/deeper evolutionary scenario as to their acquisition and sub-functionalization over vertebrate evolution. Because fibrillar collagens can be found across bilaterians, we additionally tested the relationship of these genes between gnathostomes, cyclostomes, and invertebrates, using amphioxus (*B. floridae*), tunicate (*C. intestinalis*), purple urchin (*S. purpuratus*) and leech (*H. robusta*) as representatives for invertebrate chordates, non-chordate deuterostomes, and protostomes respectively. We then inspected the genomic locations of the genes to identify conserved synteny blocks across vertebrates in further support of our phylogenies.

We were interested in identifying potential pro-ortholog families between invertebrate fibrillar collagens and their vertebrate counterparts, so we first made phylogenetic trees to test the relationship between bilaterian Clade A (Supplementary Figure S1) and Clade B (Supplementary Figure S2) genes. We did not find strong evidence for any pro-ortholog families between invertebrate and vertebrate genes, and these invertebrate sequences weakened likelihood scores across earlier-diverging nodes. This is likely due to long branch attraction occurring with the protostome and invertebrate deuterostome sequences. We thus directed our phylogenetic analyses primarily to vertebrates and the relationships between gnathostome and cyclostome genes.

**FIGURE 1:**
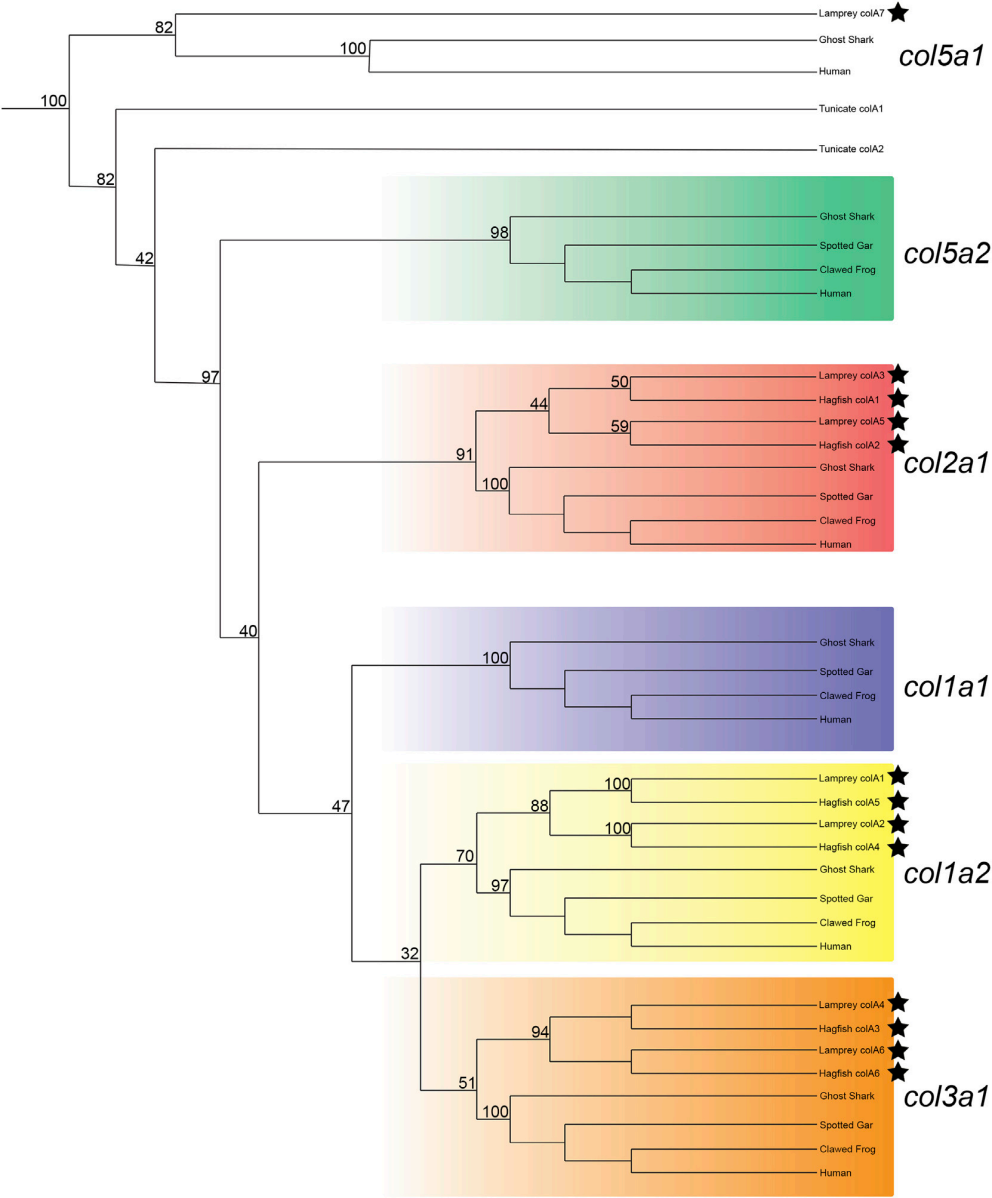
Recreated phylogenetic tree built from vertebrate and tunicate Clade A fibrillar collagen sequences. Maximum likelihood analysis scores are shown at the respective node. The human and ghost shark *col5a1* genes were chosen as the most relevant outgroup. Cyclostome genes are labelled with a black star. Original tree can be found in Supplementary Figure S3. Accession numbers for all sequences can be found in Supplementary Table S3.

After adjusting our analyses to focus specifically on vertebrates, we were able to find support for the orthology of all hagfish Clade A genes as well as six of seven lamprey Clade A genes. Both lamprey and hagfish genes correspond to three gnathostome genes: *col1a2*, *col2a1*, and *col3a1* (Figure 1). Previous work has identified *col2a1* homologs in both lamprey and hagfish (Zhang and Cohn, 2006; Zhang et al., 2006; McCauley, 2008; Ohtani et al., 2008; Ota and Kuratani, 2010; Cattell et al., 2011), and our results support these findings as well. Likewise, previous work found an additional fibrillar collagen gene in lamprey but was unable to conclusively identify its orthology (Ohtani et al., 2008), but our phylogenies support its placement as a *col1a2* ortholog. We will henceforth refer to this aforementioned gene, *colA1* in our study, as *col1a2a*. Our results likewise reveal a topology for each gene that places each lamprey and hagfish gene closer to each other than to their paralogs. These findings suggest that the number of Clade A collagens in these lineages is due to cyclostome-specific duplications. We were unable to predict the orthology of the lamprey colA7 gene, as its sequence has deviated considerably from other Clade A genes. Gene topologies like this are common in lamprey due in part to a variety of factors such as GC bias in lamprey protein-coding genes (Smith et al., 2013), long branch attraction in divergent genes (Kuraku, 2008), or these genes could even reflect a diversity of homologs no longer present in extant gnathostomes (Kuraku, 2013). Because we were unable to identify a similar gene in hagfish, *colA7* may either be a lamprey-specific duplicate or a highly divergent ortholog or paralog to an extant gnathostome gene. Our phylogenetic analyses present a topology of Clade A genes in vertebrates that suggests the loss of these genes in cyclostomes rather than their later acquisition in gnathostomes. Overall, our findings show that the last common ancestor of cyclostomes likely had five Clade A fibrillar collagens, each corresponding to the gnathostome *col1a1*, *col1a2*, *col2a1*, *col3a1*, and *col5a2* genes. The repertoire of genes found in lamprey, therefore, is the result of cyclostome-specific gene duplications and at least one gene loss, as it remains possible that *colA7* is a highly divergent ortholog of either *col1a1* or *col5a2*. We therefore designated the lamprey *colA1*, *colA2*, *colA3*, *colA4*, *colA5*, and *colA6* genes as *col1a2α*, *col1a2β*, *col2a1α*, *col3a1β*, *col2a1β*, and *col3a1α*, respectively. We likewise designated the hagfish Clade A genes with their respective lamprey ortholog.

**FIGURE 2:**
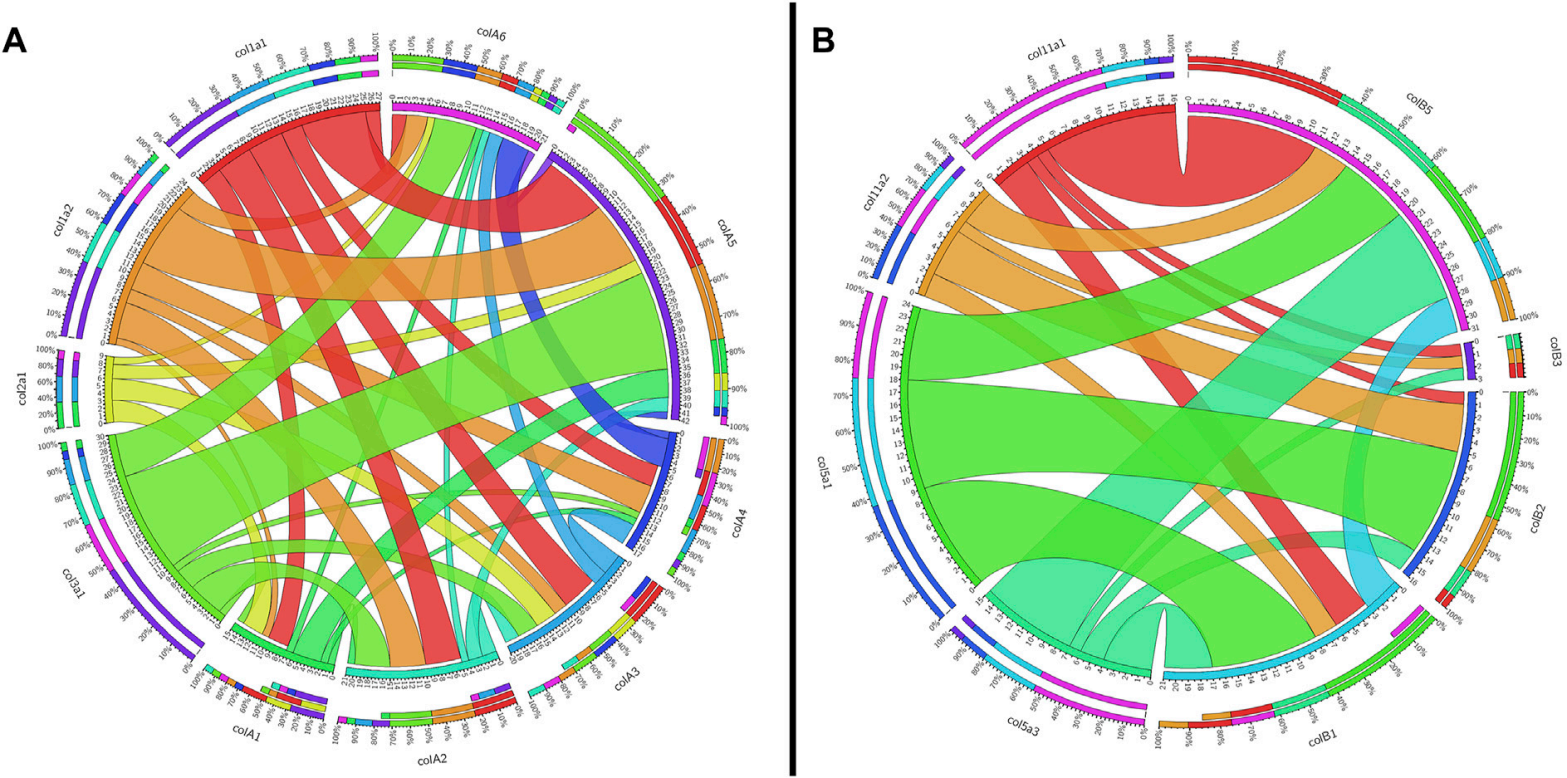
Circos plots showing the synteny between gnathostome and lamprey Clade **(A)** and Clade **(B)** fibrillar collagens. Inner circles are colored according to that gene, and they indicate the total number of linked genes and how many are syntenic with other fibrillar collagens. Outer circles show percentages of linked genes. Synteny between gnathostome genes is not shown. **(A)** Shared linkage between gnathostome and lamprey Clade A genes. All genes of interest in this plot as well as the grid used to generate it can be found in Supplementary Table S4. Because *col3a1* and *col5a2* are linked together, the latter is not shown on the plot. **(B)** Shared linkage between gnathostome and lamprey genes. All genes of interest in this plot as well as the grid used to generate it can be found in Supplementary Table S5.

To support our phylogenetic analyses for Clade A genes, we analyzed the synteny of the lamprey collagen homologs. We were able to identify *hox* clusters syntenic to each of the six major Clade A genes, a phenomenon which has been previously observed in gnathostomes (Bailey et al., 1997) (Supplementary Figure S4). Despite this, we found that the adjacent genomic locations of the lamprey collagens did not share a higher number of linked genes with their gnathostome ortholog (Figure 2A). Of the six lamprey Clade A homologs, only *colA6* shared more linked genes with its gnathostome ortholog (*col3a1*) than of the other gnathostome genes. When furthering analyzing the lamprey loci, we observed a large number of linked genes with other Clade A-containing lamprey scaffolds. These linkage clusters, however, did not correspond to those genes found near their paralog nor any particular collagen homolog. These data together suggest that the immediate genomic location of the Clade A collagens has undergone considerable rearrangement in lamprey and that the microsyntenic data for these genes alone cannot support the relationship between each lamprey and gnathostome fibrillar collagen.

**FIGURE 3:**
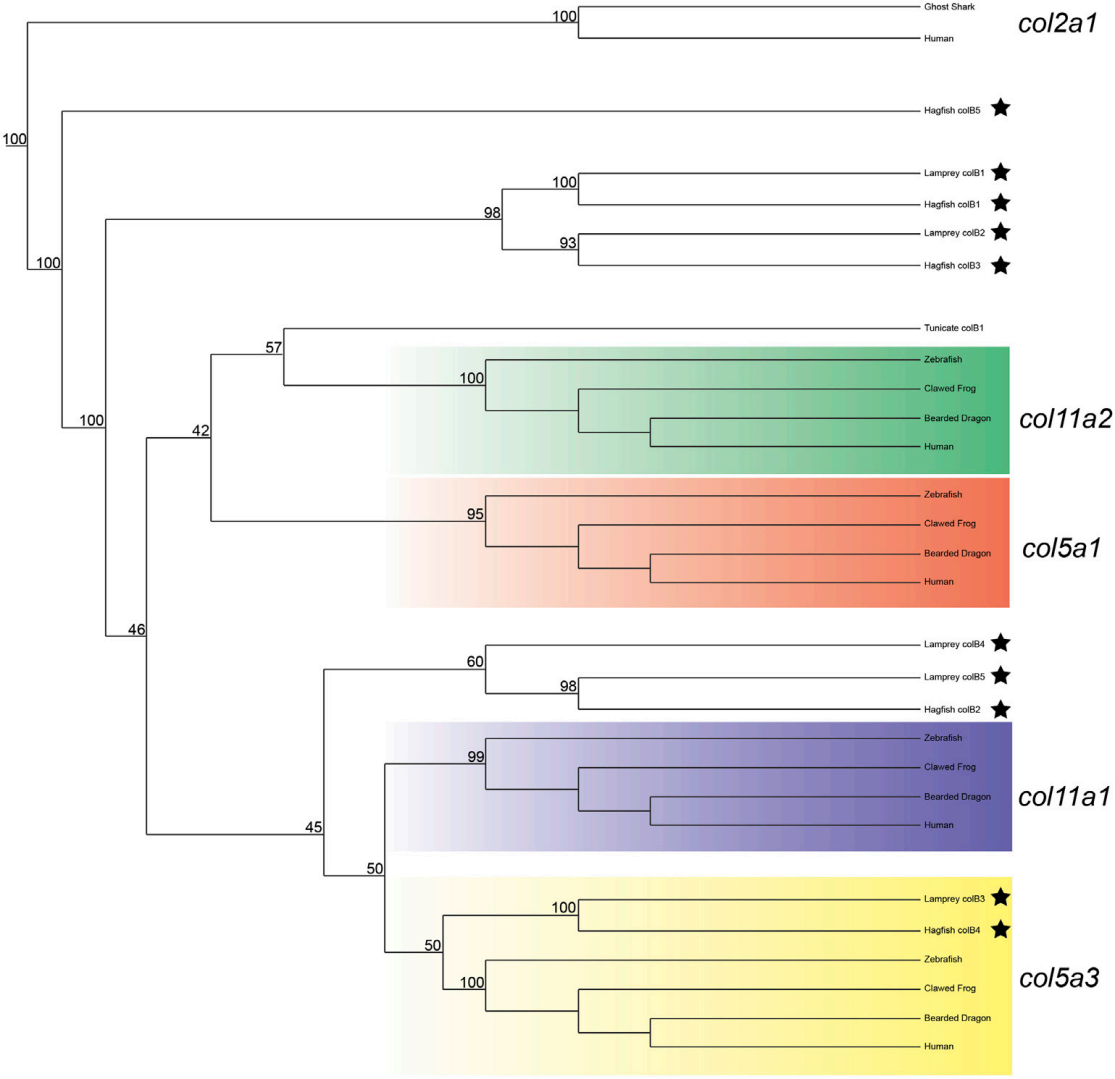
Recreated phylogenetic tree built from vertebrate and tunicate Clade B fibrillar collagen sequences. Maximum likelihood analysis scores are shown at the respective node. The human and ghost shark *col2a1* genes were chosen as the most relevant outgroup. Cyclostome genes are labelled with a black star. Original tree can be found in Supplementary Figure S5. Accession numbers for all sequences can be found in Supplementary Table S6.

We next deduced phylogenetic trees for the Clade B genes, but were unable to conclusively predict the orthology of all genes based on phylogenetics alone. We did find strong likelihood scores between complete lamprey and hagfish sequences, however, confirming that the diversity of genes found in cyclostomes was likely present in the last common ancestor. Our phylogenetic analyses provide moderate support for *colB3* as a *col5a3* ortholog, and we find some evidence for orthology of *colB4* and *colB5* within the *col11a1*/*col5a3* gene family (Figure 3). Their topology suggests that they are either related to a related gene lost in gnathostomes or are highly divergent orthologs of *col11a1*. Overall, the lamprey *colB3* gene is the only lamprey Clade B homolog which grouped with a gnathostome gene with moderate support.

Given the degree of rearrangement seen in Clade A fibrillar collagens, we were interested to see if the genomic landscape of the Clade B lamprey genes would be similar. On the contrary, we saw very little evidence of rearrangement between the homologs (Figure 2B). We additionally observed lamprey genes with a higher degree of linkage with particular gnathostome collagens. Both *colB1* and *colB2* showed considerably more linkage with *col5a1* than other genes. We found that the hagfish *colB3*, most likely the direct ortholog of lamprey *ColB2*, also showed a similar degree of linkage with *col5a1*. These together, paired with the paucity of rearrangement seen in these genes, indicate that lamprey *colB1* and *colB2* are likely *col5a1* orthologs, and they have been designated *col5a1α* and *col5a1β* respectively. While the synteny of *colB3* and *colB4* provide little insight into their relationship with gnathostome genes, the genomic arrangement of *colB5* suggests a relationship with *col11a1* and *col5a3*, as it contains a higher proportion of conserved genes with these homologs than with the other Clade B collagens. Additionally, lamprey *colB5* is distantly linked with the *colC1* gene, a similar situation seen in gnathostomes between the *col11a1* and *col24a1* genes (Exposito and Lethias, 2013). These together indicate that *colB5* is likely a *col11a1* ortholog. Combined with phylogenetic data that suggests a paralogy between *colB4* and *colB5*, we have thus designated these genes as *col11a1α* and *col11a1β* respectively. Overall, we were able to identify orthology for all lamprey Clade B genes by using a combination of phylogenetic and syntenic analyses, designating the *colB1*, *colB2*, *colB3*, *colB4*, and *colB5* genes as *col5a1α*, *col5a1β*, *col5a3*, *col11a1α*, and *col11a1β* respectively. We further designated the hagfish *colB1*, *colB2*, *colB3*, and *colB4* with their respective lamprey ortholog. Because the hagfish *colB5* is only a partial sequence, we avoided assigning it an ortholog.

**FIGURE 4:**
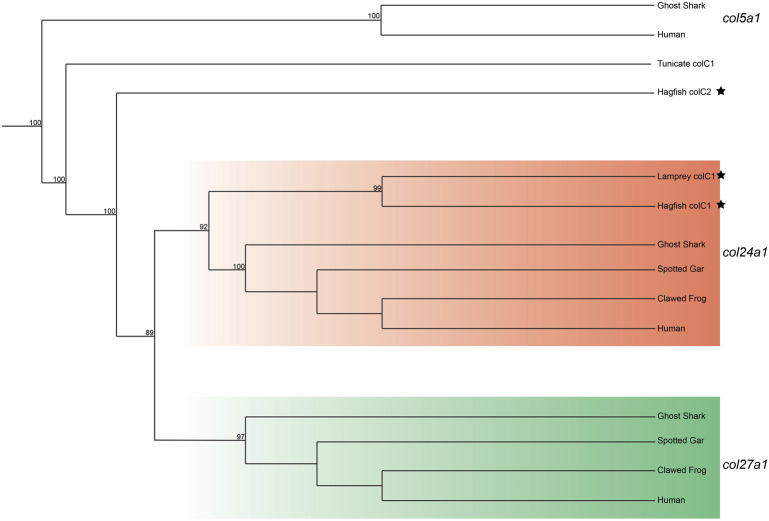
Recreated phylogenetic tree built from vertebrate and tunicate Clade C fibrillar collagen sequences. Maximum likelihood analysis scores are shown at the respective node. The human and ghost shark *col5a1* genes were chosen as the most relevant outgroup. Cyclostome genes are labelled with a black star. Original tree can be found in Supplementary Figure S6. Accession numbers for all sequences can be found in Supplementary Table S7.

We lastly built a phylogenetic tree to test the relationship of lamprey *colC1* to the gnathostome Clade C genes. The gene placed with very high certainty within the *col24a1* gene family (Figure 4). Interestingly, the second hagfish Clade C homolog placed strongly outside of both gnathostome genes. Whether the second hagfish gene represents a lineage-specific duplicate, a highly divergent *col27a1* gene, or an ancient homolog that both gnathostomes and lamprey lost, however, is unclear. Due to the very high affinity of the lamprey *colC1* as a *col24a1* ortholog and its linkage with the putative *col11a1* ortholog, however, it is likely that the last common ancestor of cyclostomes and gnathostomes had two Clade C collagens, corresponding to the *col24a1* and *col27a1* genes.

### Expression of Fibrillar Collagens in Lamprey

We next characterized the expression of these fibrillar collagens in *P. marinus* embryos through developmental stages relevant during musculoskeletal histogenesis. Given the extent of lamprey-specific innovations, we opted to focus on stages which would be most relevant for comparison with gnathostome expression. We chose Tahara stages 26 through 28 (Yutaka, 1988), corresponding to mesenchymal condensations in the oropharyngeal skeleton and the differentiation of somitic components. To confirm our observations of expression in the musculoskeletal system, we compared our results to previous reports of the lamprey musculoskeletal system, such as the developing lamprey musculature (Kusakabe et al., 2004; Kusakabe and Kuratani, 2005; Kusakabe et al., 2011), hyaline cartilage (Root et al., 2021a) [Figure S7], and mucocartilage (Cerny et al., 2010; Root et al., 2021a; Root et al., 2021b).

### Expression of Clade A Collagens

**FIGURE 5:**
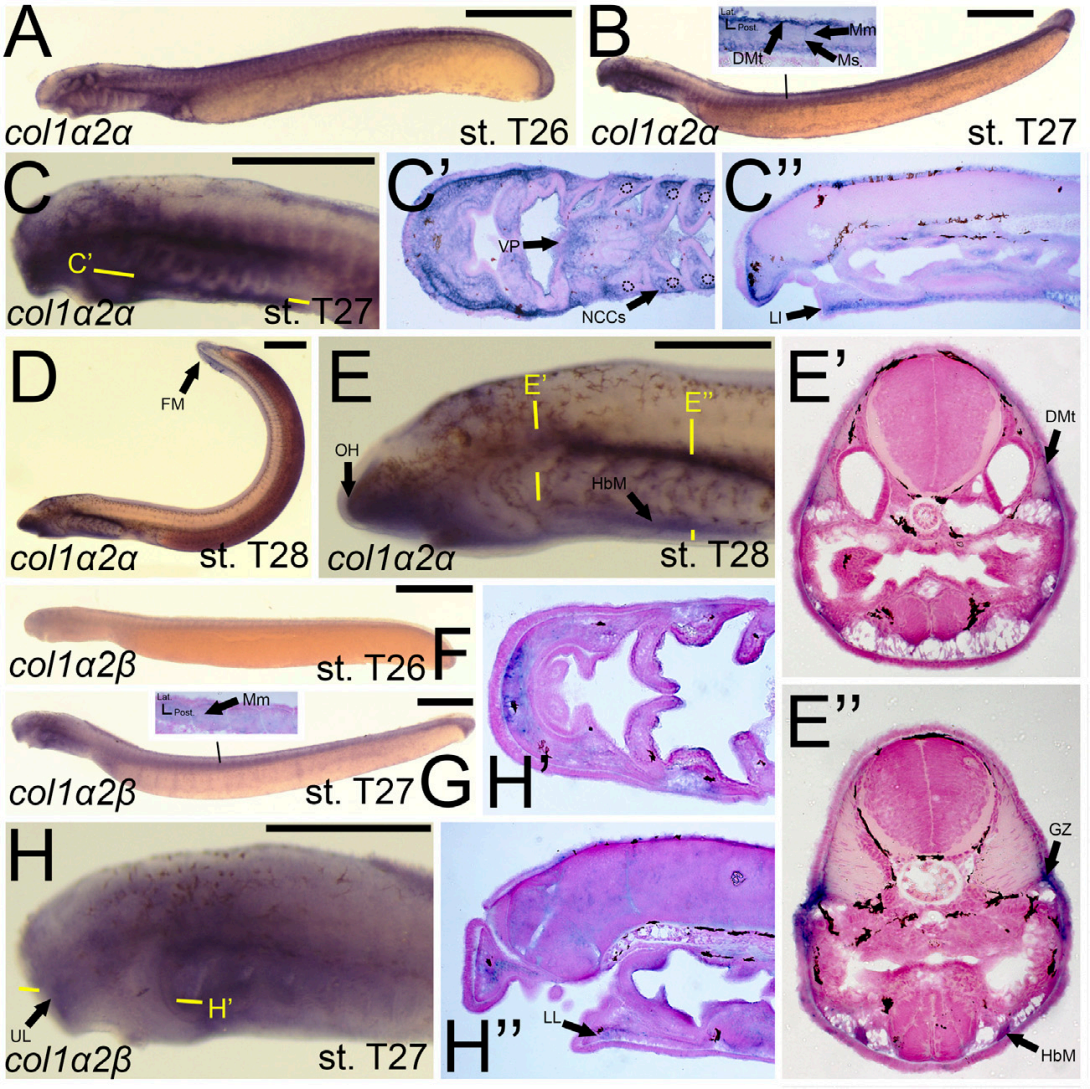
Expression of *colA1/col1a2α* and *colA2/col1a2β* in larval lamprey. All scale bars are approximately 500 μm. **(A)** *col1a2α* expression was in the cranial ganglia and throughout the head mesenchyme. **(B,C)** *col1a2α* expression was in the mesenchyme of the fin fold, lower lip, and ventral pharynx, the non-skeletogenic neural crest cells in the pharynx, the myosepta, myomeres, and the dermomyotome. Dotted circles indicate presumptive branchial arch skeleton. **(D,E)** *col1a2α* expression was in the mesenchyme of the fin fold, lower lip, and oral hood, as well as the hypobranchial musculature, the myomere growth zone, and the dermomyotome. **(F)** *col1a2β* expression was minimal at stage T26. **(G,H)** *col1a2β* expression was in the mesenchyme of the upper and lower lip, and was weak albeit present in the myomeres. Keywords: DMt: dermomyotome; FM: fin mesenchyme; GZ: growth zone; HbM: hypobranchial muscle; LL: lower lip; Mm: myomeres; Ms: myosepta; NCCs: neural crest cells; UL: upper lip; VP: ventral pharynx.

Previous work on the lamprey *col1a2* ortholog did not analyze expression at later stages (Ohtani et al., 2008), so much of our results are new for this gene. We observed a dramatic difference in expression between lamprey *col1a2* paralogs. We detected transcripts of *colA1/col1a2α* at stage T26 in the cranial ganglia, the ventral mesenchyme surrounding the endostyle, and in the somites, presumably the dermomyotome (Figure 5A). By stage T27, *colA1/col1a2α* transcripts were identified in the ectomesenchyme of the lower lip, ventral pharynx, oral hood, as well as the non-skeletogenic neural crest cells of the pharynx (Figures 5B,C,C′,C”). We also observed *colA1/col1a2α* expression in the mesenchyme of the developing fin fold as well as the dermomyotome, myomeres, and myosepta, likely presaging the myotendinous junctions (Figures 5B,D). By stage T28, this expression was limited to the mesenchyme of the oral hood and fin, the developing hypobranchial muscles, as well as the dermomyotome of the somites and the presumptive growth zone of the myomeres (Figures 5D,E,E’,E”). Conversely, we observed expression of *colA2/col1a2β* in the mesenchyme of the upper lip and lower lip at stage T27, albeit at low levels (Figures 5G,H,H”). We also observed weak expression in the developing myomeres at this stage (Figure 5G). By stage T28, expression was similar in all of the aforementioned structures as well as the fin mesenchyme.

**FIGURE 6:**
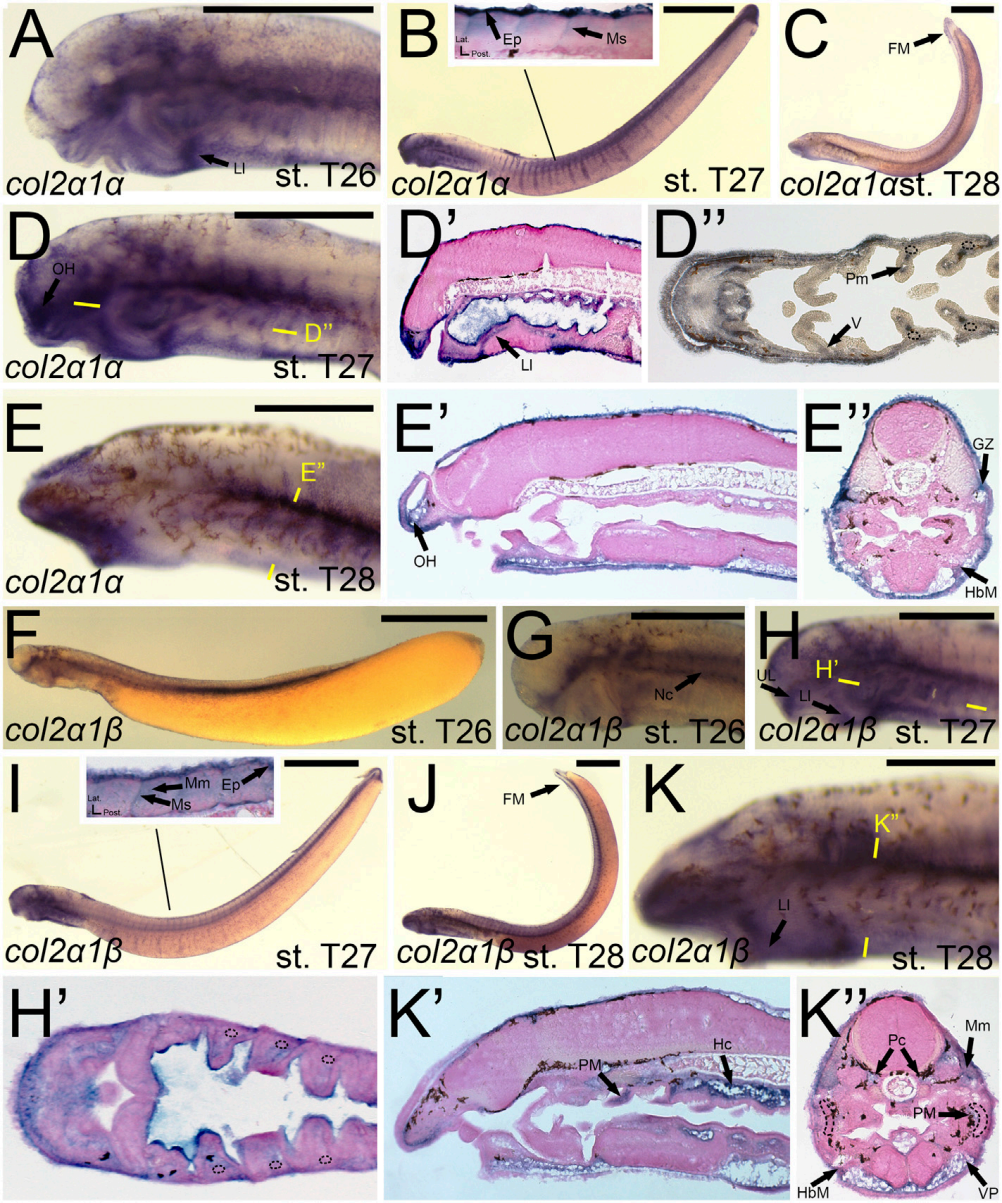
Expression of *colA3*/*col2a1α* and *colA5/col2a1β* in larval lamprey. All scale bars are approximately 500 μm. **(A)** *col2a1α* expression was in the facial ectoderm and the mesenchyme of the lower lip. **(B,D)** *col2a1α* expression was additionally found in the mesenchyme of the oral hood and lower lip as well as the lateral velum, pharyngeal muscle, epidermis, and myosepa. Dotted circles indicate presumptive branchial arch skeleton. **(C,E)** *col2a1α* expression was in the mesenchyme of the fin fold and oral hood, and was also in the hypobranchial musculature and growth zone. **(F,G)** *col2a1β* expression was in the notochord, cranial ganglia, and the mesenchyme of the upper and lower lip. **(H,I)** *col2a1β* expression was in the mesenchyme of the upper and lower lip, the pharyngeal muscle, the myomeres, epidermis, and the myosepta. **(J,K)** *col2a1β* expression was in the mesenchyme of the fin fold, ventral pharynx, and lower lip, the pharyngeal muscle, the myomeres, hypobranchial musculature, posterior hypochord, as well as the cartilages of the parachordals. Dotted circles indicate presumptive branchial arch skeleton. Keywords: Ep: epidermis; FM: fin mesenchyme; HbM: hypobranchial musculature; GZ: growth zone; Hc: hypochord; LL: lower lip; Mm: myomeres; Ms: myosepta; Nc: notochord; OH: oral hood; Pc: parachordals; PM: pharyngeal muscule; UL: upper lip; V: velum; VP: ventral pharynx.

The expression of *col2a1α* and *col2a1β* has been documented previously, and our results largely support these findings. We detected transcripts of *colA3*/*col2a1α* in the facial ectoderm and the mesenchyme of the lower lip (Figure 6A). At stage T27, we observed *colA3*/*col2a1α* expression in the epidermis and myosepta, pharyngeal muscle, as well as the mesenchyme of the lower lip, oral hood, ventral pharynx, and of the fin fold (Figures 6B,D,D′,D”). We did not, however, see expression in the hyaline cartilage of the pharynx (Figure 6D”). By stage T28, *colA3*/*col2a1α* expression in the mesenchyme and epidermis was largely maintained (Figures 6C,E,E’). We likewise noticed additional expression in the hypobranchial muscles as well as the growth zone of the myomeres (Figure 6E”). Similar to its paralog, expression of *colA5/col2a1β* matched much of what has previously been identified. We detected transcripts of *colA5/col2a1β* at stage T26 in the cranial ganglia, the mesenchyme of the upper and lower lip, and the notochord (Figures 6F,G) By T27, it was additionally found in the fin fold mesenchyme (Figure 6H,H′,I). Along the trunk, we observed a transient expression of *colA5/col2a1β* in the developing myosepta, epidermis, and myomeres (Figure 6I). At stage T28, *colA5/col2a1β* was detected in the hypochord and lower lip mesenchyme (Figure 6K,K’). Similar to the other Clade A’s, we detected expression in the myomeres and hypobranchial muscles as well as the mesenchyme of the ventral pharynx and fin fold, but we also observed distinct expression in the parachordals (Figure 6K”).

**FIGURE 7:**
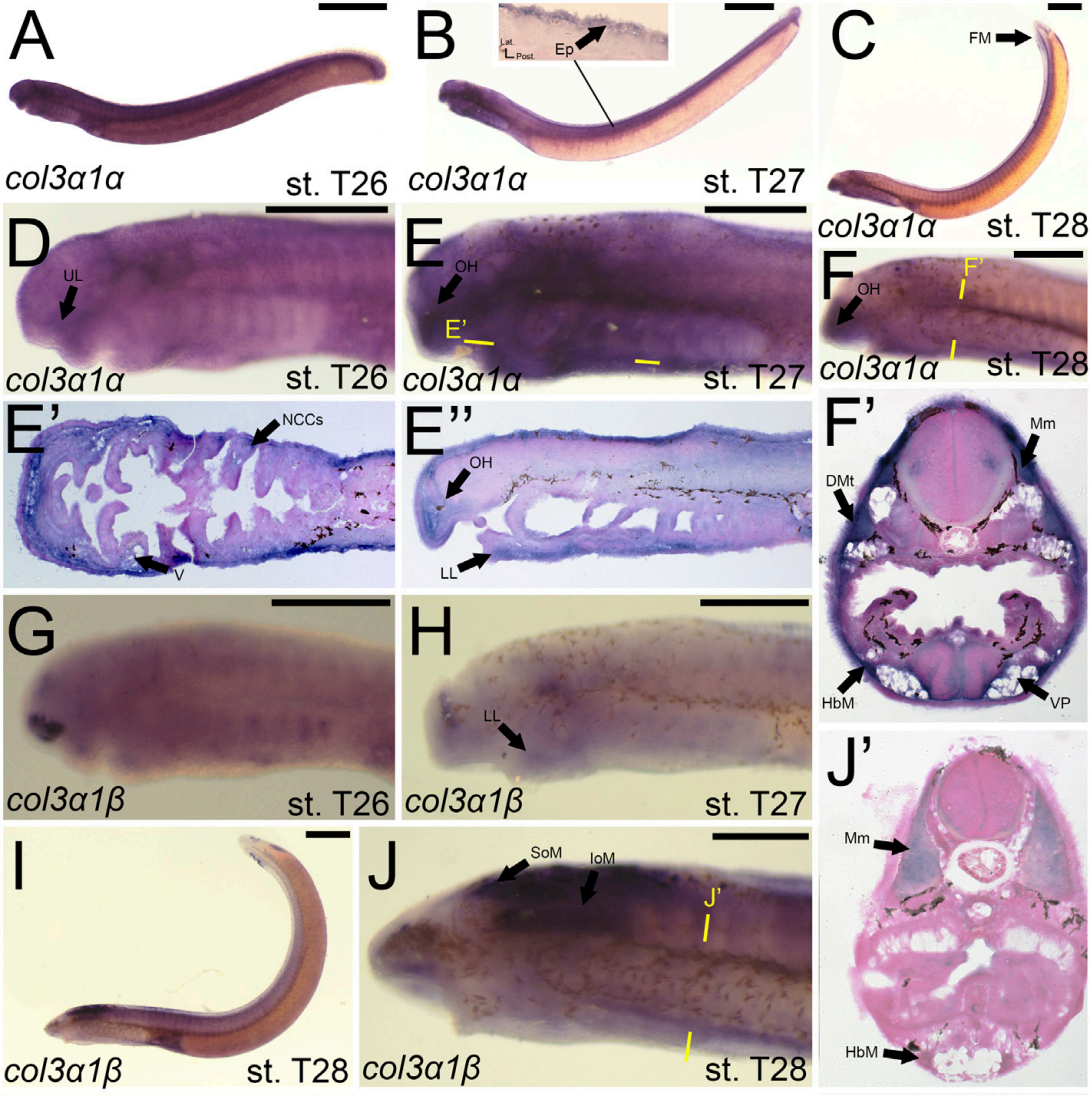
Expression of *colA6/col3a1α* and *colA4/col3a1β* in larval lamprey. All scale bars are approximately 500 μm. **(A,D)** *col3a1α* expression was in the cranial ganglia as well as the mesenchyme of the upper lip. **(B,E)** *col3a1α* expression was in the mesenchyme of the oral hood, lower lip, ventral pharynx, and velum, the non-skeletogenic neural crest cells of the pharynx, as well as the epidermis. **(C,F)** *col3a1α* expression was in the mesenchyme of the oral hood, lower lip, and ventral pharynx, the myomeres, hypobranchial musculature, and the dermomyotome. **(G)** *col3a1β* expression was in the rostral head in an unknown tissue. **(H)** *col3a1β* expression was in the forebrain and the mesenchyme of the lower lip. **(I,J)** *col3a1β* expression was in the infraoptic and supraoptic muscle populations as well as the hypobranchial musculature and myomeres. Keywords: DMt: dermomyotome Ep: epidermis; FM: fin mesenchyme; HbM: hypobranchial musculature; IoM: infraoptic muscles; LL: lower lip; Mm: myomeres; NCCs: neural crest cells; OH: oral hood; SoM: supraoptic muscles; UL: upper lip; V: velum; VP; ventral pharynx.

We noticed a difference in expression between the *col3a1* paralogs similar to that for the *col1a2* genes. Expression of *colA6/col3a1α* at stage T26 was first seen in the developing ganglia and mesenchyme of the upper lip (Figures 7A,D). We detected expression of *colA6/col3a1α* at T27 in the mesenchyme of the lower lip, oral hood, and lateral velum (Figures 7B,E,E′,E”). We also observed expression of *colA6/col3a1α* at this stage in the non-skeletogenic neural crests of the pharynx (Figure 7E’). By stage T28, expression in the cranial mesenchyme was specific to the lower lip, oral hood, and ventral pharynx, but we additionally observed *colA6/col3a1α* activity in the fin fold mesenchyme starting at this stage (Figures 7C,F,F’). Expression in the trunk continued in the hypobranchial muscles as well as the dermomyotome of the somites and the myomeres (Figure 7F’). Transcripts of *colA4/col3a1β*, conversely, were detected in the mesenchyme of only the lower lip at stage T27, but we also find expression in the developing otic vesicle and the presumptive hypophysis (Figure 7H). No muscle expression was observed at this stage. At T28, we continued to see *colA4/col3a1β* activity in the supraoptic and infraoptic muscles (Figures 7I,J). We also observed new activity in the myomeres and hypobranchial muscles (Figure 7J’).

**FIGURE 8:**
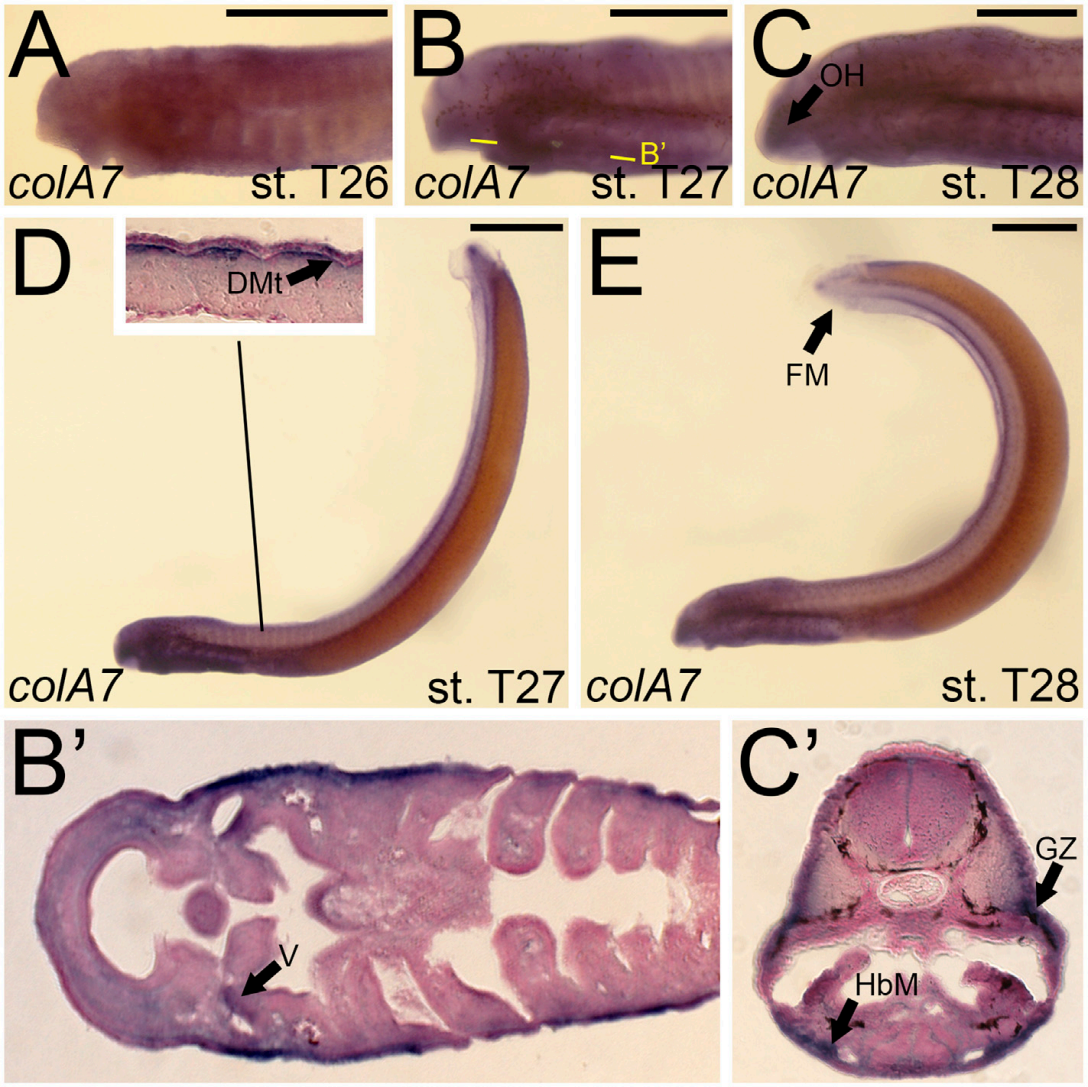
Expression of *colA7* in larval lamprey. All scale bars are approximately 500 μm. **(A)** *colA7* expression was weakly detected throughout the skin. **(B,D)** *colA7* expression was in the medial velum as well as the dermomyotome. **(C,E)** *colA7* expression was in the mesenchyme of the fin fold and oral hood, the growth zone, and the hypobranchial musculature. Keywords: DMt: dermomyotome; HbM: hypobranchial musculature; GZ: growth zone; OH: oral hood; V: velum.

Although we were unable to predict the orthology of *colA7*, we were able to design a riboprobe to characterize its expression. We detected *colA7* activity at stage T26 in the skin of the pharynx, albeit at low levels (Figure 8A). By T27, this activity expanded into the mesenchyme of the velum as well as the dermomyotome (Figure 8B,B′,D). Expression in the skin continued at this stage. *colA7* expression in the velum and pharynx abated by T28, but we detected new signal in the mesenchyme of the oral hood and fin fold as well as the growth zone of the myomeres and the hypobranchial muscles (Figure 8C,C’,E).

### Expression of Clade B and Clade C Collagens

**FIGURE 9:**
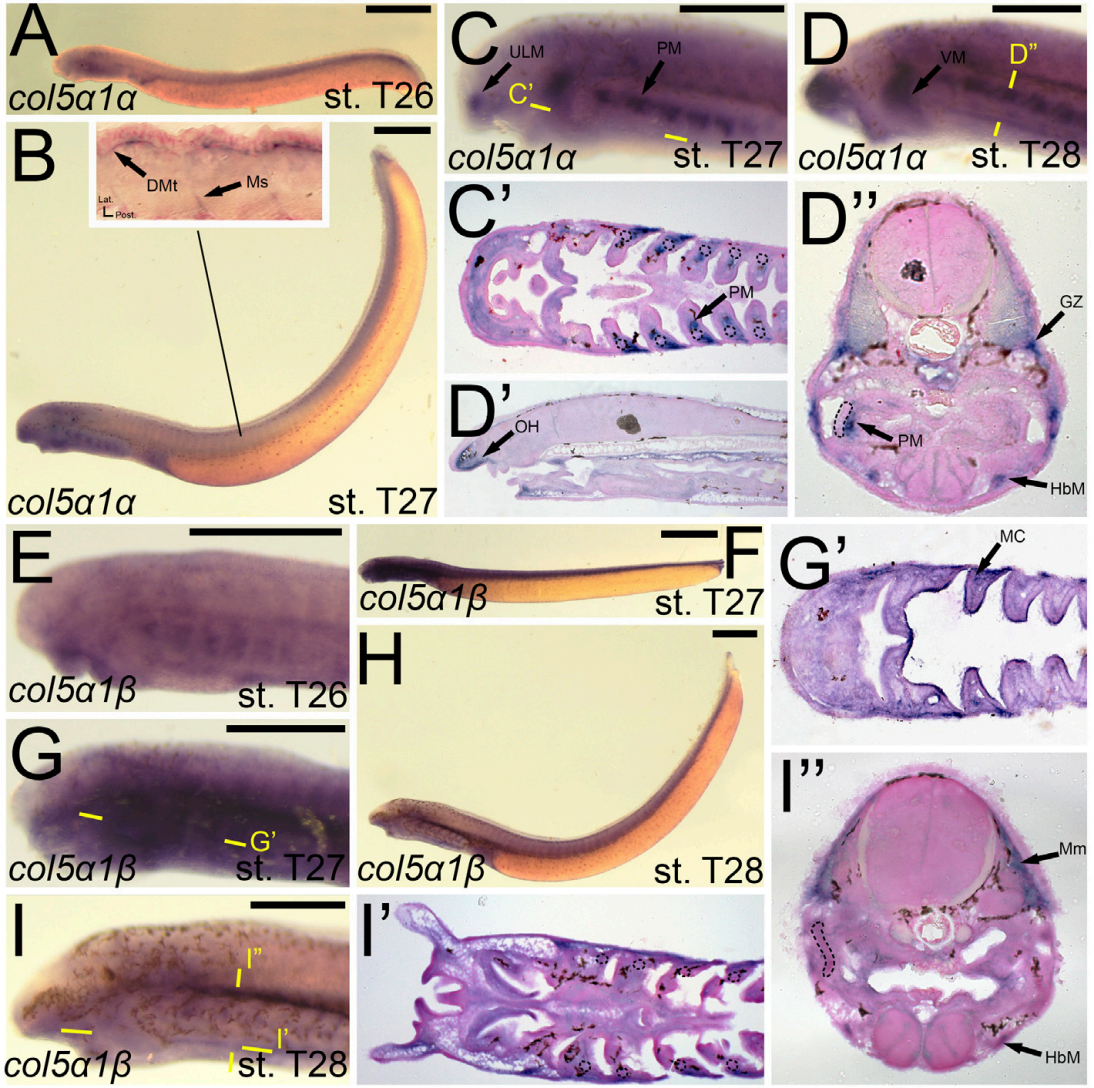
Expression of *colB1/col5a1α* and *colB2/col5a1β* in larval lamprey. All scale bars are approximately 500 μm. **(A)** *col5a1α* expression was primarily in the developing heart. **(B,C)** *col5a1α* expression was in the musculature of the pharynx, velum, and upper lip, and was also in the dermomyotome and myosepta. **(D)** *col5a1α* expression was in the mesenchyme of the oral hood, the growth zone, dermomyotome, hypobranchial musculature, and the pharyngeal muscles. Dotted circles indicate presumptive branchial arch skeleton. **(E)** *col5a1β* expression was in the oral ectoderm and throughout the pharynx albeit at low levels **(F,G)** *col5a1β* expression was in the mesodermal core of the pharynx and throughout the pharynx albeit at low levels. **(H,I)** *col5a1β* expression was in the hypobranchial musculature as well as the myomeres. Dotted circles indicate presumptive branchial arch skeleton. Keywords: DMt: dermomyotome; GZ: growth zone; HbM: hypobranchial musculature; MC: mesodermal core; Mm: myomeres; Ms: myosepta; OH: oral hood; PM: pharyngeal muscle; ULM: upper lip muscle; VM: velar muscle.

We identified expression of *colB1/col5a1α* at T27 in the upper lip muscle as well as that of the pharynx and velum (Figures 9B,C). We additionally observed *colB1/col5a1α* expression in the developing myosepta and dermomyotome (Figure 9B). By stage 28, we continued to see expression in the aforementioned regions, with additional activity noted in the mesenchyme of the oral hood, the growth zone of the myomeres, and the hypobranchial muscles (Figure 9D,D′,D”). *colB2/col5a1β* expression was considerably different from that of *colB1/col5a1α*, being identified more uniformly across the developing pharynx at stage T27 (Figure 9G,G’). At this stage, we detected *colB2/col5a1β* activity in the mesodermal core, the ectoderm of the pharynx and lower mouth, and the presumptive vascular components of pharyngeal NCC derivatives (Figure 9G’). This expression largely abates by T28, being expressed primarily in the hypobranchial musculature and the myomeres of the somites (Figure 9I,I”.

**FIGURE 10:**
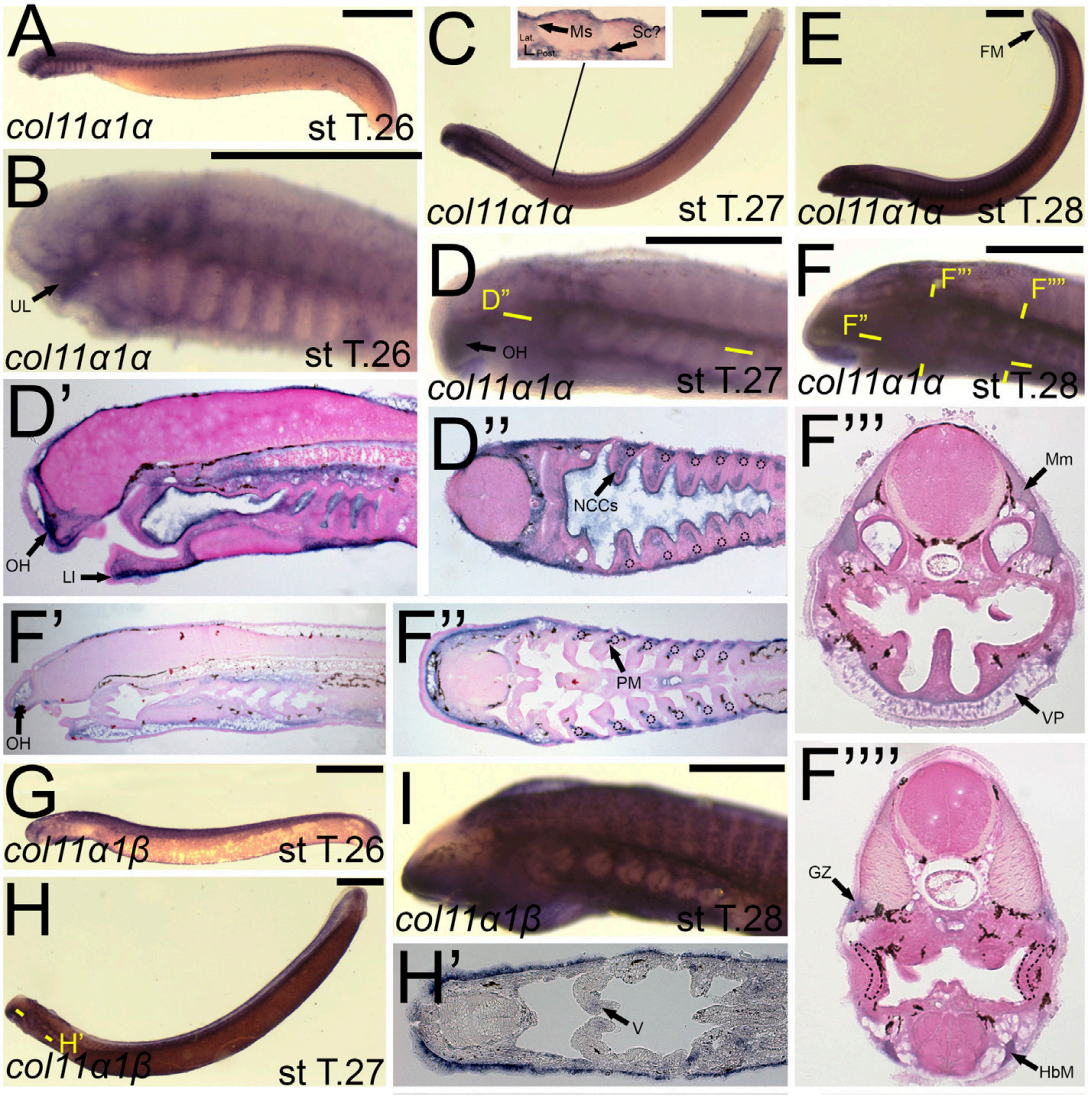
Expression of *colB4/col11a1α* and *colB5/col11a1β* in larval lamprey. All scale bars are approximately 500 μm. **(A,B)** *col11a1α* expression was in the cranial ganglia and the mesenchyme of the pharynx and upper and lower lip. **(C,D)** *col11a1α* expression was in the mesenchyme of the oral hood, lower lip, and fin fold, in the myosepta and presumptive sclerotome, and also in the non-skeletogenic neural crest cells in the pharynx. **(E,F)** *col11a1α* expression was in the mesenchyme of the fin fold, oral hood, lower lip, and ventral pharynx, the myomeres, hypobranchial musculature, pharyngeal muscles, and myomeres. Dotted circles indicate presumptive branchial arch skeleton. **(G,I)** *col11a1β* expression was superficial in the epidermis. **(H)** *col11a1β* expression was in the epidermis and in the medial velum. Keywords: Ep: epidermis; FM: fin mesenchyme; GZ: growth zone; HbM: hypobranchial musculature; LL: lower lip; Mm: myomeres; Ms: myosepta OH: oral hood; NCCs: neural crest cells; PM: pharyngeal muscle; Sc: sclerotome; UL: upper lip; VP: ventral pharynx.

The expression of *colB4/col11a1α* largely mirrored that seen in *colA1/col1a2a* at stage 26*,* being expressed in similar populations of cranial ganglia, the mesenchyme of the upper lip and pharynx, and the somites (Figures 10A,B). At T27, *colB4/col11a1α* was identified in the mesenchyme of the oral hood, lower lip, and the non-skeletogenic neural crest of the pharynx (Figures 10C,D,D′,D”). We also observed expression along the trunk and tail, visualizing *colB4/col11a1α* transcripts in the presumptive sclerotome, myosepta, and in the base of the developing fin folds (Figure 10C). At stage 28, we saw additional expression in the pharyngeal muscles, hypobranchial muscles, the myomeres and their respective growth zone, and we continued to see expression in the mesenchyme of the oral hood, ventral pharynx and fin fold (Figure 10F,F′,F″,F‴,F””). *colB5/col11a1β* was exclusively detected in the epidermis at stage 26 (Figure 10G). At T27, this expression in the epidermis is largely the same, but additional transcription was identified in the mesenchyme of the inner velum (Figure 10H,H’). *colB5/col11a1β* was additionally identified in the velar epithelium as well as that of the oral hood. By stage 28, we detected expression only in the epidermis again, with weaker activity in and around the developing gill pores (Figure 10I).

**FIGURE 11:**
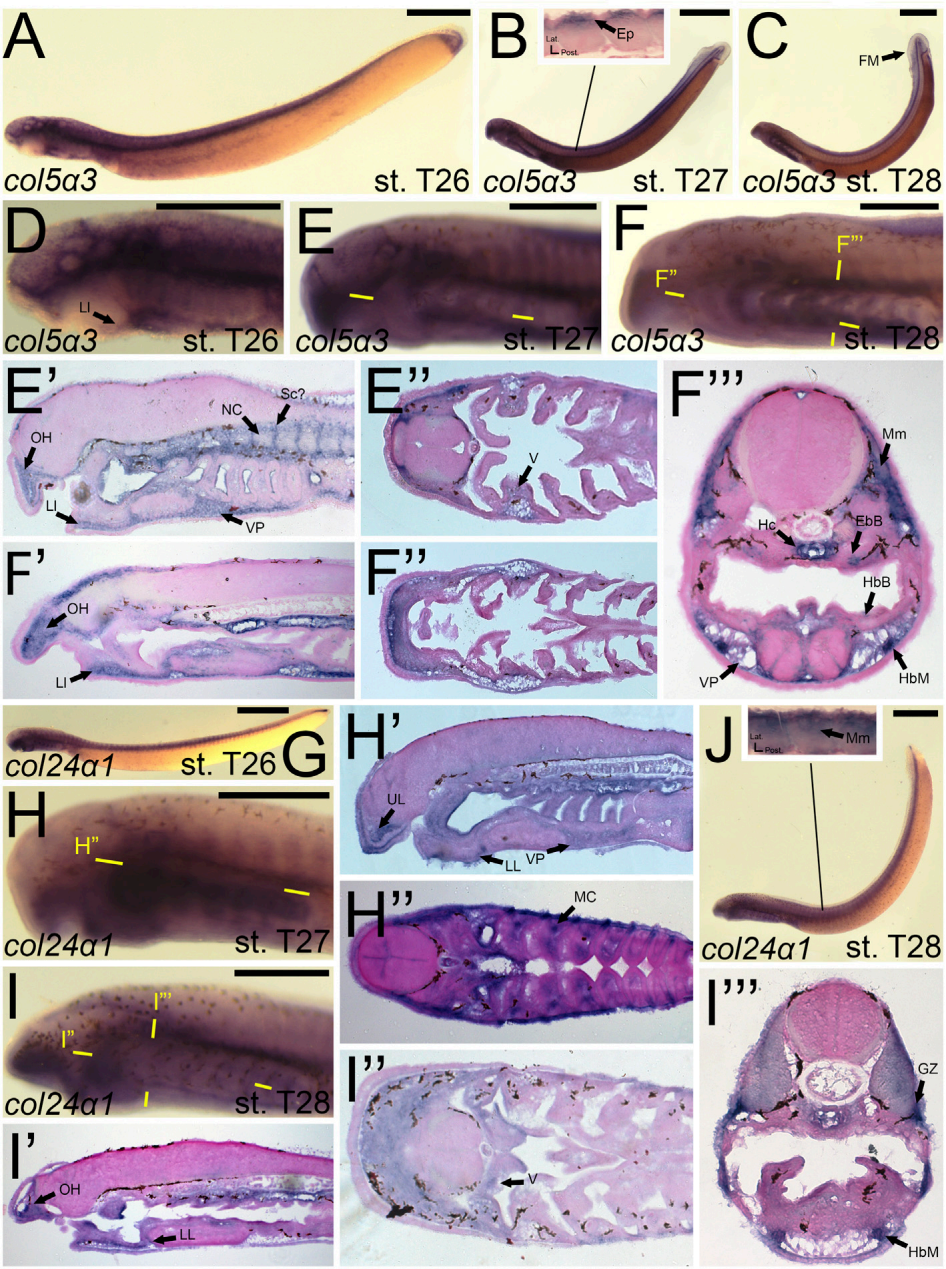
Expression of *colB3/col5a3* and *colC1/col24a1* in larval lamprey. All scale bars are approximately 500 μm. **(A,D)** *col5a3* expression was in the cranial ganglia and the mesenchyme of the upper and lower lip and ventral pharynx. **(B,E)** *col5a3* expression was in the mesenchyme of the oral hood, lower lip, ventral pharynx, lateral velum, fin fold, and presumptive sclerotome, as well as the notochord and epidermis. **(C,F)** *col5a3* expression was in the mesenchyme of the aforementioned areas as well as the hypobranchial musculature, myomeres, hypochord, and the cartilages of the epibranchial bars and hypobranchial bars. Dotted circles indicate presumptive branchial arch skeleton. **(G,H)** *col24a1* expression is in the mesodermal core of the pharyngeal arches as well as the mesenchyme of the upper and lower lip as well as the ventral pharynx. **(I,J)** *col24a1* expression is in the mesenchyme of the oral hood, lower lip, ventral pharynx, and velum, and is also in the hypobranchial musculature, growth zone, and myomeres. Keywords: Ep: epidermis; EpB: epibranchial bars; FM: fin mesenchyme; GZ: growth zone; HbB: hypobranchial bars; HbM: hypobranchial musculature; Hc: hypochord; LL: lower lip; NC: notochord; OH: oral hood; MC: mesodermal core; Mm: myomeres; Sc: sclerotome; UL: upper lip; V: velum; VP: ventral pharynx.

We first found *colB3/col5a3* activity at stage T26 in the developing ganglia and mesenchyme of the lower lip (Figures 11A,D). *colB3/col5a3* was identified at T27 in the various pre-skeletogenic populations of mesenchyme, specifically the oral hood, lower lip, ventral pharynx, fin fold, as well as the lateral velum (Figures 11B,E,E′,E”). We also observed expression at this stage in the presumptive sclerotome and epidermis (Figure 11B,E’). At stage 28, *colB3/col5a3* skeletal activity in the sclerotome was absent, but we did observe new expression in the hypochord and epibranchial bars and hypobranchial bars that are dorsal and ventral to the hyaline cartilage of the pharynx (Figure 11F,F′,F″,F‴). Expression in the oral hood, lower lip, fin fold, and ventral pharynx are similar to that seen at T27 (Figure 11F’,F‴). We additionally noted *colB3/col5a3* in the hypobranchial musculature as well as the anterior myomeres and growth zone (Figure 11F”’).

*colC1/col24a1* was expressed at both T26 and T27 in the mesenchyme of the upper and lower lip (Figures 11G,H,H′,H”). We also observed *colC1/col24a1* activity in the mesodermal core of the pharyngeal arches, the otic vesicle, and the pharyngeal pouches (Figure 11H”). By stage 28, transcripts were further identified in the mesenchyme of the oral hood, ventral pharynx. and throughout the velum as well as the hypobranchial musculature, myomeres, the dermomyotome, and the growth zone (Figure 11I”).

## Discussion

This study represents, to the best of our knowledge, the most comprehensive single study of fibrillar collagens and their gene expression in a vertebrate. Additionally, it is the first analysis of major and minor fibrillar collagens in a cyclostome. Future studies with collagens in lamprey connective tissues should use histochemical stains like picrosirius red to corroborate results, although this method still needs to be refined in lampreys (Supplementary Figure S8). Despite this, we used *in situ* hybridizations and sectioning to find multiple instances of fibrillar collagen co-expression across the musculoskeletal system, most dramatically in the hypobranchial musculature and the skeletogenic mesenchyme of the oral hood and fin fold. Our gene expression analyses support previous work for each gene which appear to be exclusive to specific groups. Overall, our findings are an important step in our understanding of fibrillar collagens and their evolution across vertebrates.

### Gnathostomes and Lamprey Have Largely Conserved the Ancestral Collagen Repertoire

Our phylogenetic and syntenic analyses revealed distinct orthologs between the gene families in cyclostomes and gnathostomes as well as lineage-specific paralogs. Structural comparisons of the genes between these lineages, however, is unlikely to provide any new insights. Paired with macrosyntenic data, we can hypothesize the evolution of these gene families and its deviation in both cyclostomes and gnathostomes. The presence or absence of certain fibrillar collagens could provide insight into larger evolutionary trends.

Our phylogenetic analyses suggest that the last common ancestor of cyclostomes and gnathostomes had five Clade A fibrillar collagens. While gnathostomes have maintained all five genes, hagfish have likely lost both col1a1 and col5a2 whereas lamprey have lost at least one of these genes, the uncertainty stemming from whether *colA7* is a highly divergent ortholog of one of these genes or a lamprey-specific duplicate of a different gene. Cyclostomes overall have more Clade A genes in part to three sets of paralogs, corresponding to *col1a2*, *col2a1*, and *col3a1*. There are considerable differences in expression between each set of paralogs. Interestingly, the developing skeleton of the fin expresses six of the seven Clade A genes. The loss of either *col1a1* or *col5a2* has interesting implications for the evolution of cyclostomes. There are no expression domains for *col5a2* which are gene-specific, meaning that its function may be largely redundant. As seen with the loss of *col5a3* and *col11a2* in certain gnathostome taxa, it is possible that the gene was lost before the divergence of cyclostomes, its function being compensated by other genes. Conversely, the loss of *cola1* may be more direct with a loss of morphology, being associated with the absence of mineralization in extant cyclostomes. Material studies have demonstrated the importance of type I collagen, composed of *col1a1* proteins, in the formation of hydroxyapatite crystals during mineralization (Tomoaia and Pasca, 2015). Recent paleontological evidence now suggests a possible relationship between lamprey and hagfish and with conodonts, an ancient jawless vertebrate with mineralized teeth (Goudemand et al., 2011; Terrill et al., 2018). If conodonts were stem cyclostomes, then the loss of mineralization in modern lampreys and hagfishes may have coincided with the loss of *col1a1* and other genes involved in the mineralization process. Both scenarios, however, depend on whether *colA7* is orthologous to *col5a2a* or *col1a1* or if it is a lamprey-specific duplicate instead.

Our results suggest that gnathostomes have maintained much of the ancestral repertoire of Clade B genes, but losses in this family are more common than in Clade A. Lamprey and hagfish no longer have the *col11a2* gene but have duplicated the *col5a1* gene and possibly *col11a1* as well. Compared to Clade A, we see more variation in Clade B genes across gnathostomes, with several lineages having lost either *col5a3* or *col11a2*. Similar to Clade A genes, we see considerable sub-functionalization between these paralogs, but their expression domains do not deviate beyond what we have seen in fibrillar collagens. These genes are a minor component of the collagenous ECM, so it is possible that their functions are similar enough that they can be compensatory for one another. Overall, the function of Clade B collagens has been largely maintained across extant vertebrates, being involved in most components of the musculoskeletal system, but they have drifted in function across lineages.

Our phylogenetic analyses suggest that the last common ancestor of cyclostomes and gnathostomes had two genes in the Clade C family, and the *col27a1* gene was likely lost in lamprey. The extent of expression found in *col24a1* is therefore interesting in contrast to gnathostomes, which is limited mostly to developing bone in the musculoskeletal system (Koch et al., 2003; Wang et al., 2012; Duran et al., 2015). The difference in expression between the gnathostome Clade C genes and their lamprey counterpart could be due in part to the sub-functionalization of these genes in the gnathostome lineage. Therefore, the lamprey *col24a1* gene may reflect an ancestral domain of the gene in connective tissues, somewhat mirroring the range of expression seen in *col1a2* or *col1a1*.

### Fibrillar Collagens in Skeletal Muscle Are Largely Conserved Across Vertebrates

While fibrillar collagens are a less abundant component of the muscular ECM, their importance in these connective tissues remains an important area of study (Rowe, 1981; Eghbali and Weber, 1990; Rescan, 2019; Sleboda et al., 2020). Similar to expression patterns seen in gnathostomes, lamprey deploy fibrillar collagens in this connective tissue. An interesting pattern to note, however, is the contrast in lamprey expression between the medial myotome and lateral dermomyotome as well as between the anterior and posterior myotomes. Furthermore, the number of fibrillar collagen genes expressed in the musculature is also different between lamprey and gnathostomes. Jawed vertebrates only utilize type V collagen (*col5a1* and *col5a3* genes) in muscular connective tissue whereas lamprey use most of their Clade B genes to some extent. The abundance of lamprey fibrillar collagen genes in musculature is exemplified in the developing hypobranchial muscles, where *col1a2a, col2a1a, col2a1b, col3a1a, col3a1b, col5a1a*, *col5a1b*, *col5a3*, *col11a1a*, and *col24a1* are all co-expressed.

The exact role of fibrillar collagens during muscle development in vertebrates is unclear. It is thought that the lateral and dorsoventral ends of the dermomyotome are a source of muscle fibers for secondary myogenesis, also known as stratified hyperplasia, and that these muscle fibers will have a distinct composition compared to the primary myofibers (Barresi et al., 2001; Georgiou et al., 2016; Chu et al., 2018). The presence of specific collagens in the medial or lateral domains of the dermomyotome indicate that, by these developmental stages, they are likely beginning to differentiate between the dermis and these secondary muscle fibers. These secondary muscle fibers are thought to contribute differentially to fast and slow-twitch muscle fibers, each having distinct collagen compositions and thus different mechanical properties (Kovanen et al., 1984; Nakamura et al., 2003). It is also possible, however, that these collagens are associated with the immature myoblasts of the dermomyotome, which control the rate at which differentiation and hyperplasia occur (Denetclaw et al., 2001; Ordahl et al., 2001; Steinbacher et al., 2006; Rescan et al., 2013). Further work must be done, therefore, to elucidate the role of fibrillar collagens in myogenesis and muscle homeostasis in lamprey.

The contrast in fibrillar collagens in the anterior and posterior myomeres may be attributed to the unique patterns of muscle migration in the lamprey head which have been previously described (Kusakabe and Kuratani, 2005; Kusakabe et al., 2011), but not all migratory muscles have similar collagen expression. Of particular interest is the infraoptic muscle, which did not express any collagens visualized in this study. It is thought that this muscle does not have a clear homolog in gnathostomes (Kusakabe et al., 2011), and its functions and mechanics remain unknown. Additionally, we observed a difference in fibrillar collagens between the anterior myomeres and the hypobranchial musculature, both of which are migratory muscle populations. This would suggest that there are some mechanical differences between the lamprey migratory muscles.

Taken together, our findings suggest that vertebrate fibrillar collagens have a conserved role during early myogenesis, with type V collagen being predominantly myogenic in distribution. They are expressed in both the early myotome and dermomyotome, and likely contribute to different myogenic populations. The main differences between lamprey and gnathostome fibrillar collagens in the developing muscles are the presence of type II, type XI, and type XXIV collagen in lamprey, where they are thoroughly co-expressed in the hypobranchial muscles and the myomeres and their respective growth zone. Whether this is due to a possible role in maintaining myoblasts or the development of secondary fibers, however, is uncertain. The function of collagens in the muscular system remains poorly understood, and further work is necessary to elaborate on the activity of these genes in the tissues. Overall, fibrillar collagens have a minor but conserved role in vertebrate musculature.

### The Lamprey Collagenous Skeleton Differs Greatly From Gnathostomes

It has been noted for nearly two centuries that the lamprey larval and adult skeletons were very different from those seen in gnathostomes (Müller, 1834; Schneider, 1879; Parker, 1883; Johnels, 1948), but only in more recent years has a more descriptive genetic and molecular approach been possible. Among these observations were the identification of novel lamprey proteins like lamprin and pharymprin which are differentially expressed in the larval and adult skeletons (Wright et al., 1983; Robson et al., 1993; McBurney et al., 1996; Yokoyama et al., 2019). Additionally, we have learned more about the differences in gene expression and histology between the gnathostome-like hyaline cartilage of the larval trabeculae and branchial skeleton and that of mucocartilage, the avascular connective tissue scattered throughout the anterior head skeleton that has several characteristics of articular cartilage (Johnels, 1948; Cerny et al., 2010; Cattell et al., 2011; Yao et al., 2011; Root et al., 2021b). Recent work has shown that the lamprey larval skeleton expresses multiple *Lectican* homologs in distinct skeletal populations (Root et al., 2021a), an interesting feature which supports previous hypotheses that the lamprey larval skeleton may have as many as six cartilage types (Cattell et al., 2011).

Our results show that lamprey mucocartilage is rather homogenous for fibrillar collagens, particularly so in the oral region and fin fold, with each co-expressing five shared fibrillar collagens (*col1a2a*, *col2a1a*, *col3a1a*, *col5a3*, *col11a1a*). Additionally, the oral hood expresses *col5a1a* and *col24a1* whereas the fin fold expresses *col1a2b* and *col2a1b*.

A rather intriguing find was the absence of fibrillar collagens in the branchial arch cartilages throughout all developmental stages noted. Previous studies have identified type II collagen in these arches at later developmental stages as well as the adult skeleton (Zhang et al., 2006), but it was unclear whether *col2a1* orthologs were present in the prechondrogenic condensations or early chondrocytes of these arches in *P. marinus*. Our findings suggest that neither major nor minor fibrillar collagens are present in the early differentiation and condensation of sea lamprey branchial cartilage. This contrasts with collagen expression data across all metazoan cartilages, where Clade A collagens are present (Tarazona et al., 2016). Furthermore, type II collagen has been documented in the branchial cartilages of the arctic lamprey *Lethenteron camtschaticum* during early development (Ohtani et al., 2008), meaning that it is likely that *P. marinus* have lost type II collagen in these cartilages during these developmental stages. This sea lamprey deviation is an important insight into vertebrate chondrogenesis, as *col2a1* is thought to be essential for normal cartilage development.

**FIGURE 12:**
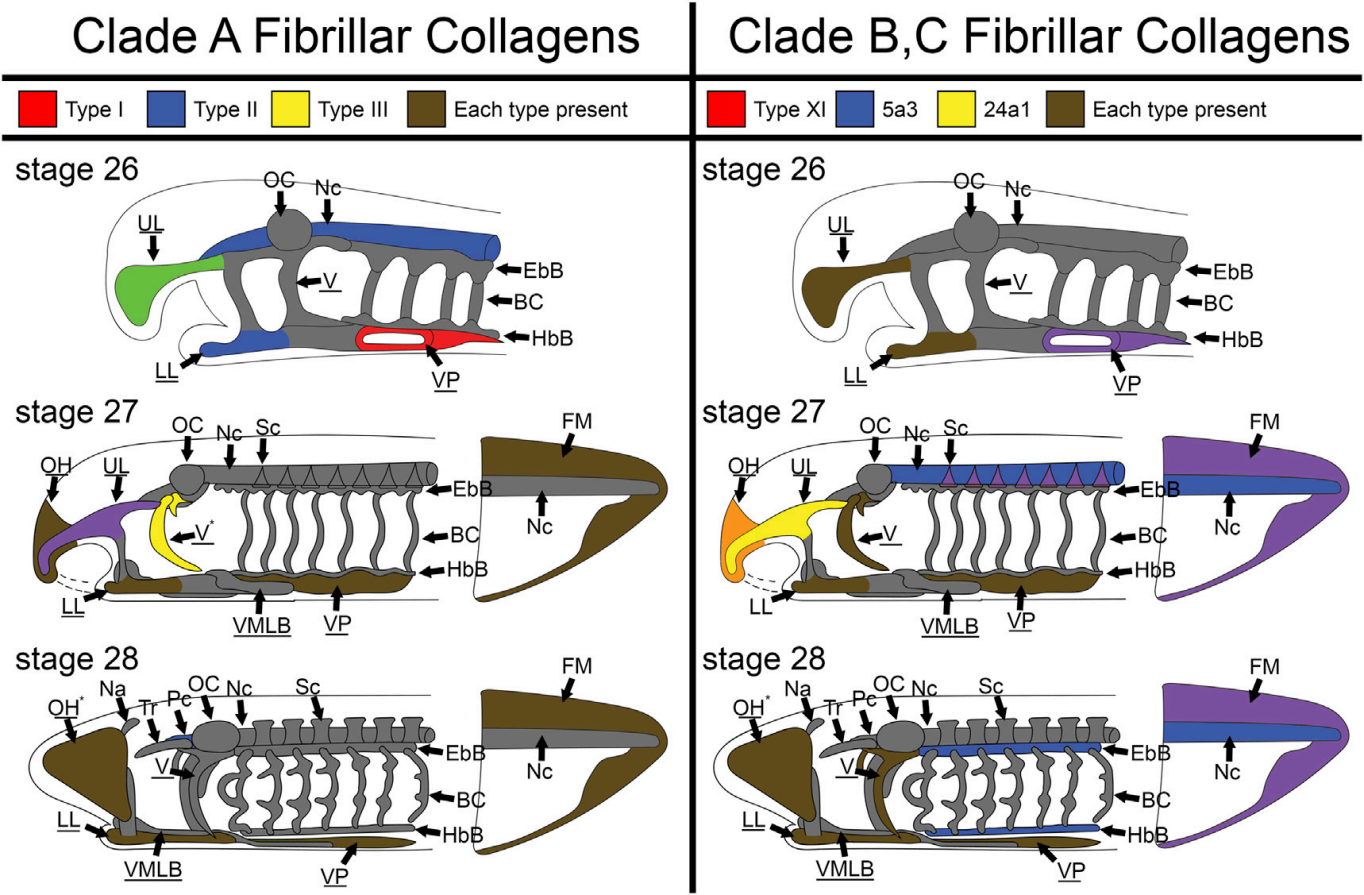
Skeletal heterogeneity of fibrillar collagens in larval lamprey during early development. For stage T26, only the head skeleton is shown, whereas both head and fin skeleton are shown for T27 and T28. Colors indicate specific subfamilies for each Clade. Minor components of the skeleton for each clade (*colA7* and *col5a1α* for Clade A and Clade B respectively) are indicated by an asterisk near the tissue where they are expressed instead of a color. Non-primary colors signify a combination of collagen subfamilies, and gray indicates no fibrillar collagens. Underline keywords indicate skeletal populations which are considered mucocartilage. Although fibrillar collagens are largely ubiquitous the oral region and ventral pharynx later in development, they are entirely absent from the branchial cartilages, otic capsule, nasal capsule, and trabeculae throughout the stages observed, and are also particularly lacking in similar cartilages like the parachordals, epibranchial bars, and hypobranchial bars. Fibrillar collagens are also heterogeneous throughout the posterior mucocartilage. Keywords: BC: branchial cartilages; EbB: epibranchial bars; FM: fin mesenchyme; HbBL: hypobranchial bars; LL: lower lip; Na: nasal capsule; Nc: notochord; OC: otic capsule; OH: oral hood; Pc: parachordals; Sc: sclerotome; Tr: trabeculae; UL: upper lip; V: velum; VMLB: ventromedial longitudinal bar; VP: ventral pharynx.

Although *col2a1* is the most well-attested fibrillar collagen in the gnathostome cartilaginous skeleton and *col1a1* in the bony skeleton, several other collagens are known to have transient roles in the development of these structures. It is therefore not surprising that the larval sea lamprey skeleton expresses a variety of fibrillar collagens during development (Figure 12). What is important to note, however, is the spatial differences in these collagens throughout the lamprey skeleton during these stages of development. Lamprey have sub-functionalized cartilage types in different regions of the skeleton, and we have found distinct collagen deployments throughout the head and fin skeleton that support these observations. Overall, these findings suggest that the ancestral vertebrate cartilage extracellular matrix utilized multiple fibrillar collagens, but sea lamprey have innovated upon this program to create distinct skeletal types and no longer require them for initial hyaline development.

### Fibrillar Collagens Were Multifunctional in the Ancestral Vertebrate Musculoskeletal System

Fibrillar collagens have been previously documented in invertebrate chordates like tunicates and amphioxus, with most genes being expressed in the oral cirri, notochord, the tail bud, and the musculature (Wada et al., 2006; Zhang and Cohn, 2006; Jandzik et al., 2015). While the expansion of this gene family would have likely contributed to sub-functionalization amongst cell types in vertebrates, our results in lamprey, paired with gnathostome literature, suggest the contrary. We posit that the general function of the fibrillar collagen family has remained relatively unchanged over the course of chordate evolution, with a conserved role in the development and maintenance of connective tissues.

It remains of great interest why so many fibrillar collagens have been maintained in vertebrates if the majority of them are co-expressed across cell types to some extent. Our current understanding of gene duplication posits that redundant genes are eventually purged from the genome, but there may also be long-term benefits to this redundancy (Wapinski et al., 2007; Kuzmin et al., 2020). We see differences in specificity between orthologs in both lineages, with type II and type XI collagen being predominantly expressed in skeleton in gnathostomes and type V collagen being almost entirely expressed in muscle in lamprey. These together would suggest that has been some change of function for these genes. The extracellular matrix is a complicated meshwork of dozens of different types of proteins, and it is therefore possible that each collagen provides distinct structural support or even modulations to cell signaling. However, it is also possible that co-expression of fibrillar collagens helps overcome rate-limited transcription for such abundantly-needed ECM proteins, meaning that these collagens are redundant and interchangeable to some extent, at least in the musculoskeletal system. Whether these differences in co-expression are due to qualitative or quantitative differences is an important aspect of fibrillar collagen evolution that may help us further understand their roles in development.

The evolution of the vertebrate lineage coincided with multiple events of genomic expansion, meaning that most gene families are larger than their invertebrate counterparts. Understanding how these genes sub-functionalized and supported the rise of new tissue types is important for our understanding of vertebrate musculoskeletal evolution. Our results suggest that the function of fibrillar collagens was largely established before the divergence of extant vertebrates, having important roles throughout the developing skeleton and musculature. The overlap we see in these genes across the cell types is likely due to their piecemeal contribution to the mechanics and signaling of connective tissues, regardless of the progeny. The total diversity of fibrillar collagen genes present in vertebrates may be due to either redundancy or subtle sub-functionalization, with differences in their distribution in cell types being attributed to differences in signaling or mechanics. We therefore suggest that the main functions of fibrillar collagens were likely established before the divergence of modern vertebrates and that the evolution of the vertebrate musculoskeletal system involved upstream regulatory differences to the deployment of fibrillar collagens, allowing ancestral connective tissues to diversify in form and function.

## Methods

### Isolation of Lamprey and Hagfish Collagen Homologues

Lamprey collagen sequences were tiled from transcriptomic reads of Tahara st. 26.5 embryos and adult oral disc tissue that were previously gathered and submitted to GenBank (Wheeler et al., 2007). Hagfish collagen sequences were tiled from transcriptomic reads of Bashford Dean st 28–30 embryos generously provided by Juan Pascual Anaya at the Universidad de Malaga and were submitted to GenBank (Wheeler et al., 2007). To identify lamprey and hagfish sequences, we used zebrafish fibrillar collagen protein sequences to query *P. marinus* and *E. burgeri* transcriptomes using tBLASTN, collecting the top ten reads for each to ensure that zebrafish-specific biases were minimized. These reads were translated and searched against gnathostome genomes (*D. rerio*, *C. milii*, *H. sapiens*) to confirm whether they were Clade A, Clade B, or Clade C genes. To confirm that our lamprey transcriptomic sequences represented all possible fibrillar collagen genes, we used UCSC’s genome browser and the BLAT tool (Kent, 2002) to identify the locations in the lamprey genome where potential fibrillar collagens could be located. These sequences were additionally tested using BLASTp to identify which fibrillar collagen clade they likely corresponded to, letting us organize the primary sequences and remove partial sequences before our analyses.

Full-length sequences from these files were used for our phylogenetic and syntenic analyses. For *in situ* hybridizations for *colA3/col2a1α* and *colA5/col2a1β*, primers were designed from lamprey genomic sequence to amplify conserved exon sequences, which were cloned into the pJet1.2 vector. For the remainder of the lamprey genes, 500-550bp regions from transcriptomic sequences were selected and ordered as fragments in pUC57-amp vector from Synbio Tech©.

### Phylogenetic Analyses

For our phylogenetic analyses, full-length gnathostome fibrillar collagens were chosen to best represent the breadth of gnathostome genomic and morphological diversity as well as select invertebrates where both Clade A and Clade B genes could be identified, and these sequences would be compared against lamprey and hagfish fibrillar collagen. Full-length human fibrillar collagen protein sequences were obtained from NCBI (Wheeler et al., 2007) and used to identify additional gnathostome and invertebrate collagen protein sequences using BLASTP (Altschul et al., 1990). GenBank accession numbers used are available in Tab. S3-S7. In the case of sturgeon (*Acipenser oxyrhinchus*) sequences, zebrafish collagen protein sequences were used to find transcripts using tBLASTN. These sequences were then translated and searched against multiple gnathostome genomes to confirm their identity. We then aligned all sequences for each analysis using the PROBALIGN (Roshan and Livesay, 2006) program on CIPRES (Miller et al., 2011) servers. We used a gap open penalty of 20 and a gap extension penalty of 1. To determine the optimal substitution model for our phylogenetic analysis, we used ProtTest v3.4.2 (Abascal et al., 2005) In the ProtTest settings, we allowed for invariant sites, empirical frequencies, and BIONJ-defined tree topology to determine our ideal model. For our main Clade A test, a JTT + I + G model was calculated with a log likelihood score of -111,200.671 under Akaike Information Criterion (AIC). For our main Clade B test, a JTT + I + G model was calculated with a log likelihood score of -97422.354 under AIC. For our Clade C test, a JTT + I + G model was calculated with a log likelihood score of -76554.941 under AIC. For our phylogenetic analysis, we used maximum likelihood analyses using RAxML-HPC2 Workflow on CIPRES servers. Using the aforementioned parameters provided by ProtTest, our likelihood scores were bootstrapped with 1,000 trees for each test to derive a consensus tree. For our Clade A and Clade C analyses, we used an outgroup of *col5a1* homologues from human and ghost shark. For our Clade B analysis, we used an outgroup of *col2a1* homologues from human and ghost shark. Our consensus trees were lastly visualized using FigTree v1. 4.4 (Rambaut, 2014).

### Synteny Analyses

For our linkage analyses, we used peptide sequences of gnathostome fibrillar collagen genes from our phylogenetic analyses and found their respective genomic location using UCSC’s Genome Browser and the BLAT tool (Kent, 2002). For our estimate of gnathostome linkage groups, we looked at the loci of dog (*C. lupus familiaris*), chicken (*G. gallus*), zebrafish (*D. rerio*), and clawed frog (*X. tropicalis*), as these species have well-assembled genomes with chromosomes/scaffolds long enough to detect distant linkage. For our macrosynteny analyses, we gathered peptide sequences of gnathostome fibrillar collagens on NCBI and found their respective genomic location using UCSC’s Genome Browser and the BLAT tool (Kent, 2002) as well as ENSEMBL (Yates et al., 2020). We used sequences of human (*Homo sapiens*), clawed frog (*Xenopus tropicalis* or *Xenopus laevis*), spotted gar (*Lepisosteus oculatus*), zebrafish (*Danio rerio*), and elephant shark (*Callorhinchus milii*) to reconstruct the ancestral arrangement of genes around the fibrillar collagen loci. Because the lamprey genome has undergone considerable rearrangement, we did not limit our search for linked genes based on distance alone, instead identifying ≈27–30 genes immediately 5′ and 3′ of each collagen locus when possible. We annotated each gene according to the genome browser’s prediction. We then color-coded the lamprey genes based on which gnathostome fibrillar collagen it was mostly linked to. Due to the strong GC bias of lamprey protein coding sequences and the possibility of distinct duplicates, we included all homologs of a gene family at a particular locus. We then attempted to find similar linked genes around the hagfish collagen loci using the aforementioned methods. Due to the current condition of the hagfish genome assembly, we were unable to find any scaffolds that were long enough to effectively search. To better visualize our synteny results for the manuscript, we generated a Circos plot using the online version of Circos Table Viewer (Krzywinski et al., 2009), creating a non-reciprocal plot to show linked genes across gnathostome and lamprey fibrillar collagens.

### Embryo Collection and Staging

Embryos for *in situ* hybridization were obtained from adult spawning-phase sea lampreys (Petromyzon marinus) collected from Lake Huron, MI, and kept in chilled holding tanks as previously described (Nikitina et al., 2009). Embryos were staged according to the method of Tahara (Yutaka, 1988), fixed in MEMFA (Mops buffer, EGTA, MgSO4, and formaldehyde), rinsed in Mops buffer, dehydrated into methanol, and stored at –20 °C.

### *In Situ* Hybridization

Riboprobes were made for anti-sense fragments using SP6 RNA polymerase. Sequences for probes and genes are available upon request. In our experience, full-length P. marinus riboprobes, or riboprobes generated against untranslated regions of P. marinus transcripts, give higher background than short riboprobes against coding sequences. We believe that this is because lamprey noncoding sequences, especially 3′ UTRs, often have an excessive GC-repeat content, causing corresponding riboprobes to hybridize nonspecifically to off-targets. To mitigate this, we made short 550-bp riboprobes against coding regions and used a high-stringency hybridization protocol (Cerny et al., 2010; Square et al., 2016). Key parameters of this protocol include post-hybridization washes at 70 °C and the use of a low-salt, low-pH hybridization buffer (50% formamide; 1.3× SSC, pH 5.0; 5 mM EDTA, pH 8.0; 50 μg/ml tRNA; 0.2% Tween-20; 0.5% CHAPS; and 100 μg/ml heparin).

Histology, Histochemistry, and Sectioning.

After *in situ* hybridization, embryos were postfixed in 4% paraformaldehyde/PBS (4°C, overnight), rinsed in PBS, cryo-protected with 15% sucrose/PBS, embedded in 15% sucrose, 20% gelatin/PBS (37°C, overnight), and 20% gelatin/PBS (37°C overnight), frozen in liquid nitrogen, and mounted in OCT compound (Miles). Cryo-sections of 14 μm were collected on Super Frost Plus slides (Fisher Scientific), counterstained using Nuclear Fast Red (Vector Laboratories), and dehydrated and mounted in DPX (Fluka) (Jandzik et al., 2014). For lamprey *colA3/col2a1α* and *colB5/col11a1β* stage T27 sections, *in situ* hybridizations were performed on 10 μm paraffin sections which were deparaffinized in Histoclear immediately beforehand. For these sections, proteinase K, hybridization, and color development times were shortened. Picrosirius staining was performed on 14 μm paraffin sections which were deparaffinized in Histoclear and rehydrated, stained in 1% picrosirius red for 30 min, and differentiated twice in 1% acetic acid for 5 min each before being dehydrated and mounted.

### Imaging

Whole-mount *in situ* hybridized P. marinus embryos and larvae were photographed using a Carl Zeiss Axiocam MRc5, Carl ZeissDiscovery V8 dissecting microscope, and Axiovision 4.9.1 software. Sections were photographed using a Carl Zeiss Imager A2 compound microscope.

## Data Availability

The datasets presented in this study can be found in online repositories. The names of the repository/repositories and accession number(s) can be found in the article/Supplementary Material.
