# Supplementary figures and images for: A Comprehensive Analysis of Fibrillar Collagens in Lamprey Suggests a Conserved Role in Vertebrate Musculoskeletal Evolution
Source: Front Cell Dev Biol. 2022 Feb 15;10:809979. doi: 10.3389/fcell.2022.809979 (PMC8887668; doi:10.3389/fcell.2022.809979)

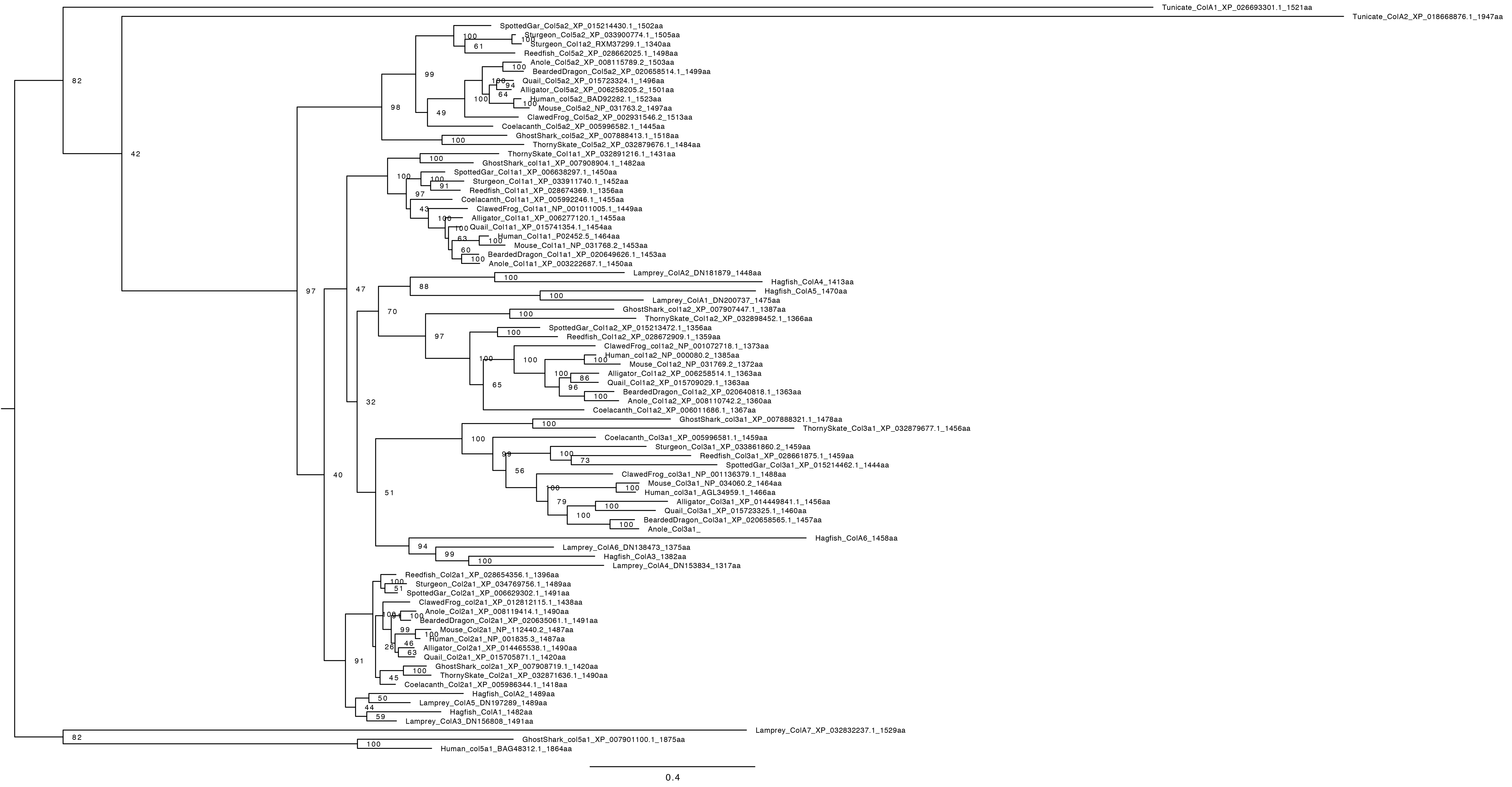

Supplement: Supplementary file 1 [file Image3.jpeg]

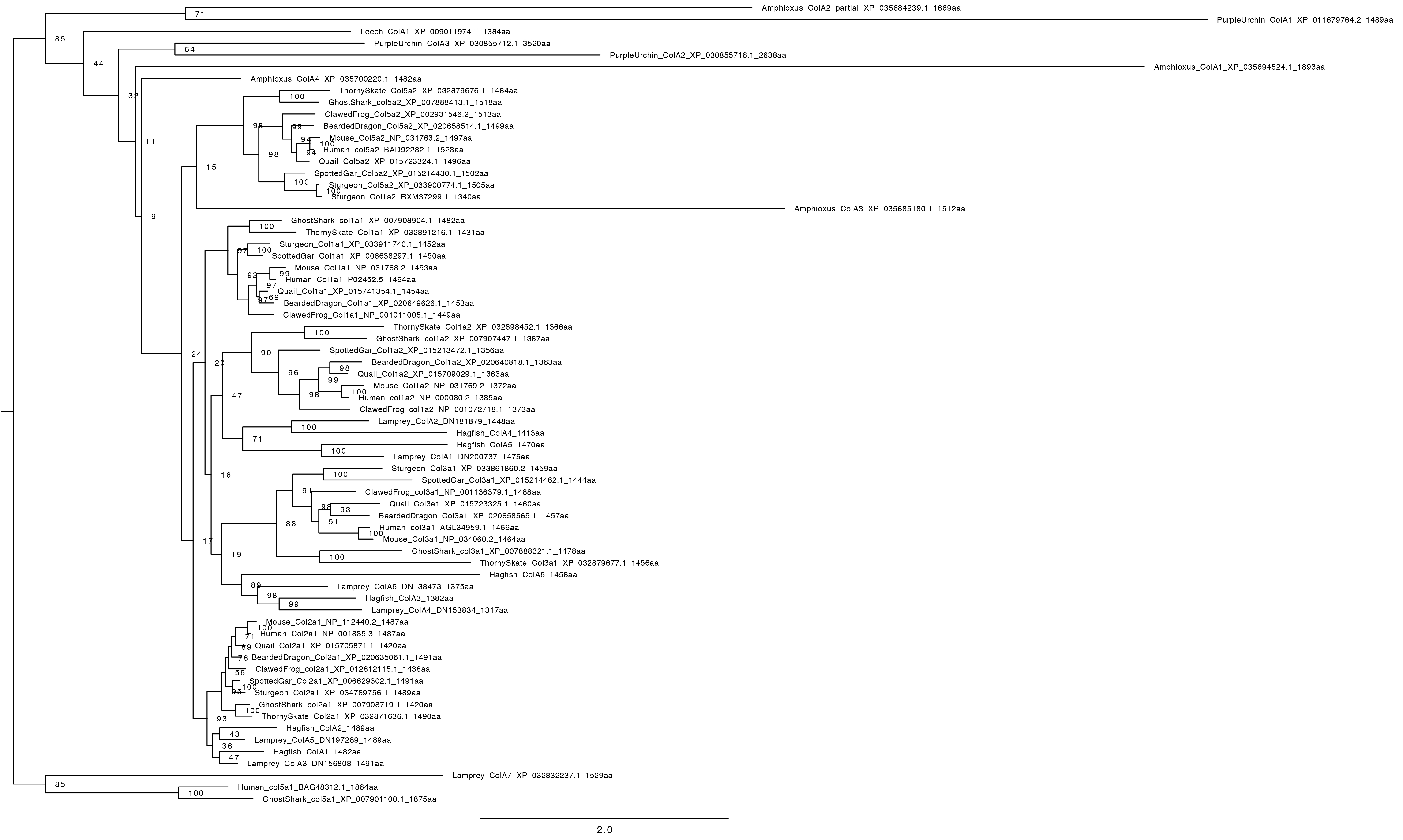

Supplement: Supplementary file 3 [file Image1.jpeg]

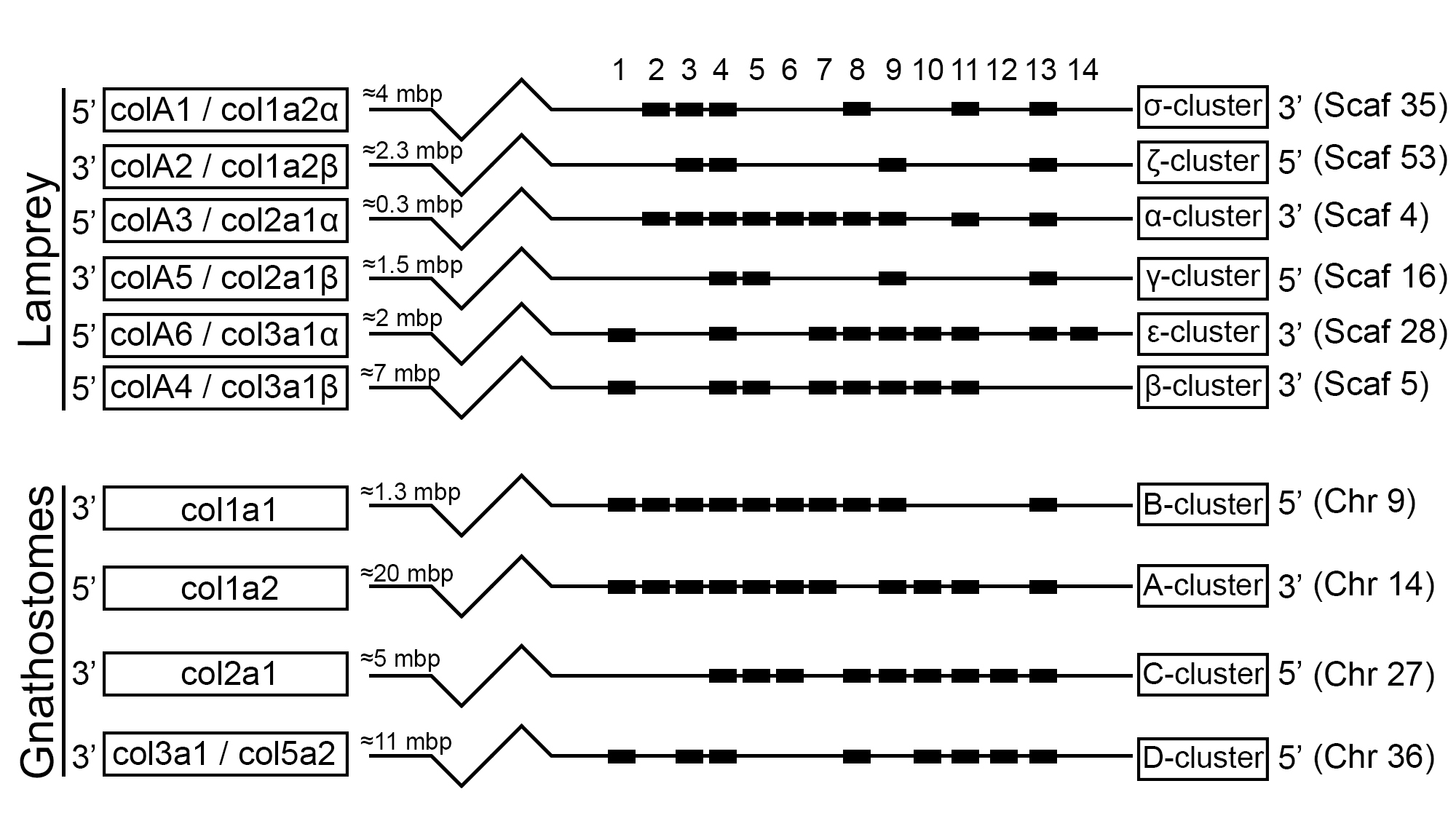

Supplement: Supplementary file 4 [file Image4.jpeg]

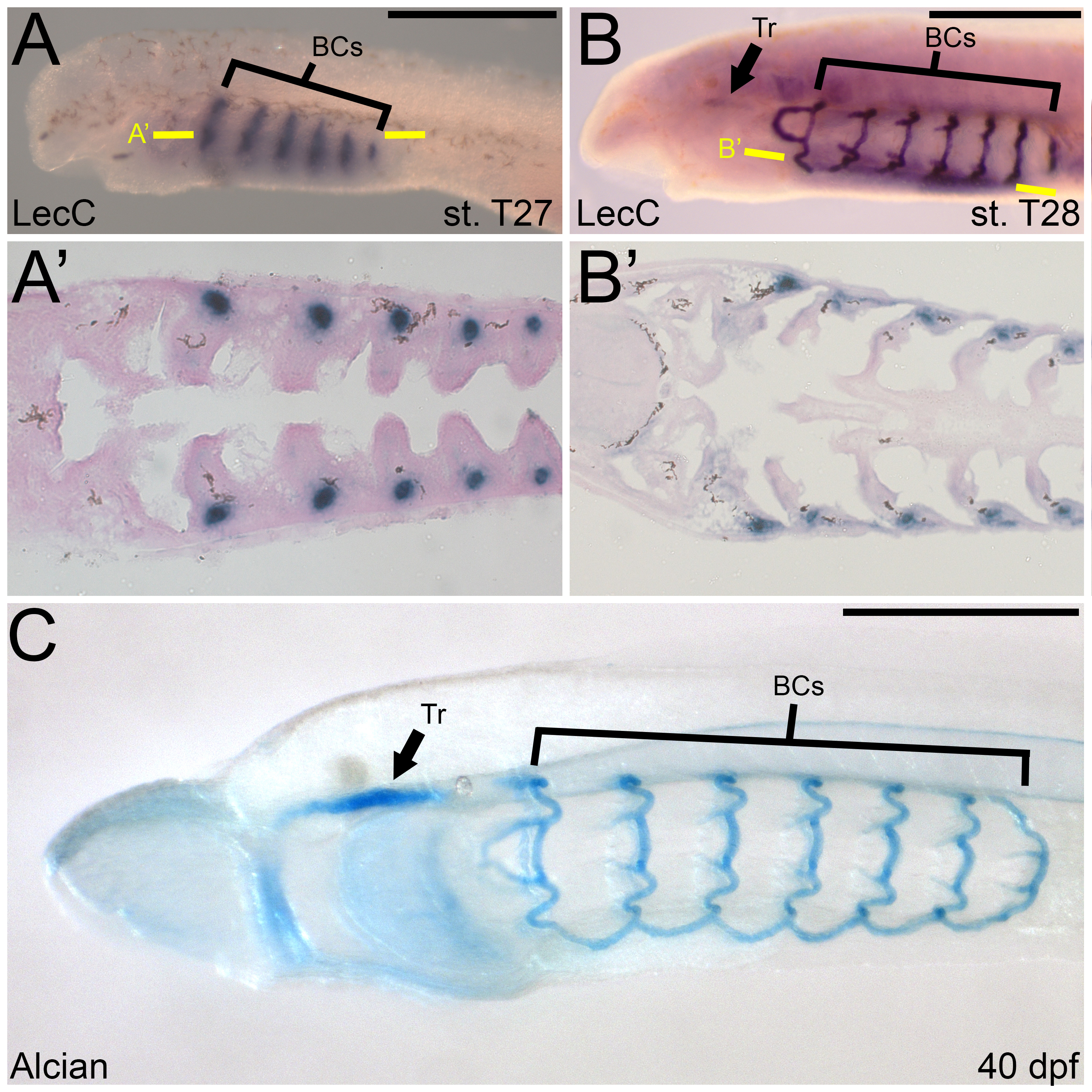

Supplement: Supplementary file 5 [file Image7.jpeg]

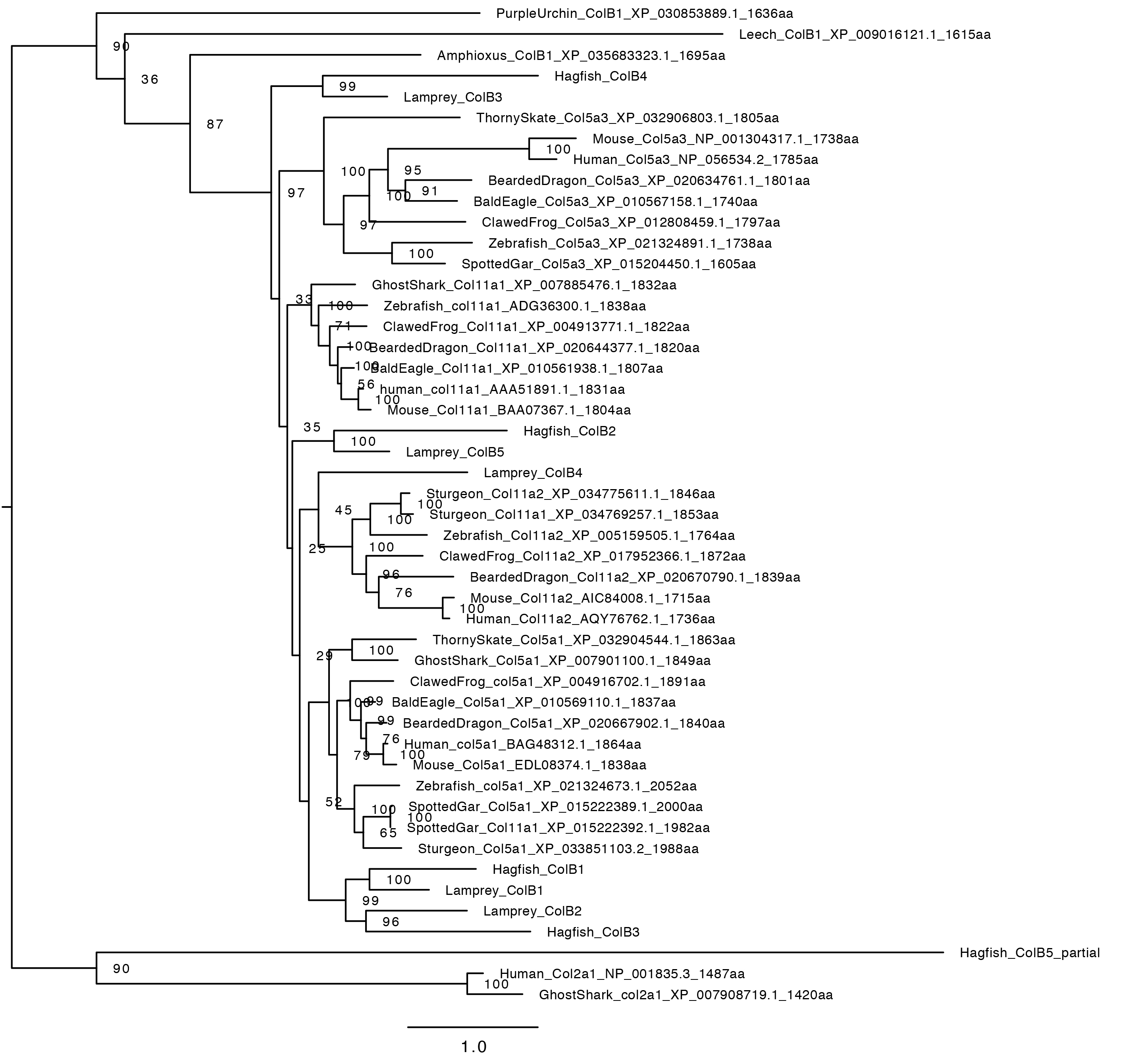

Supplement: Supplementary file 7 [file Image2.jpeg]

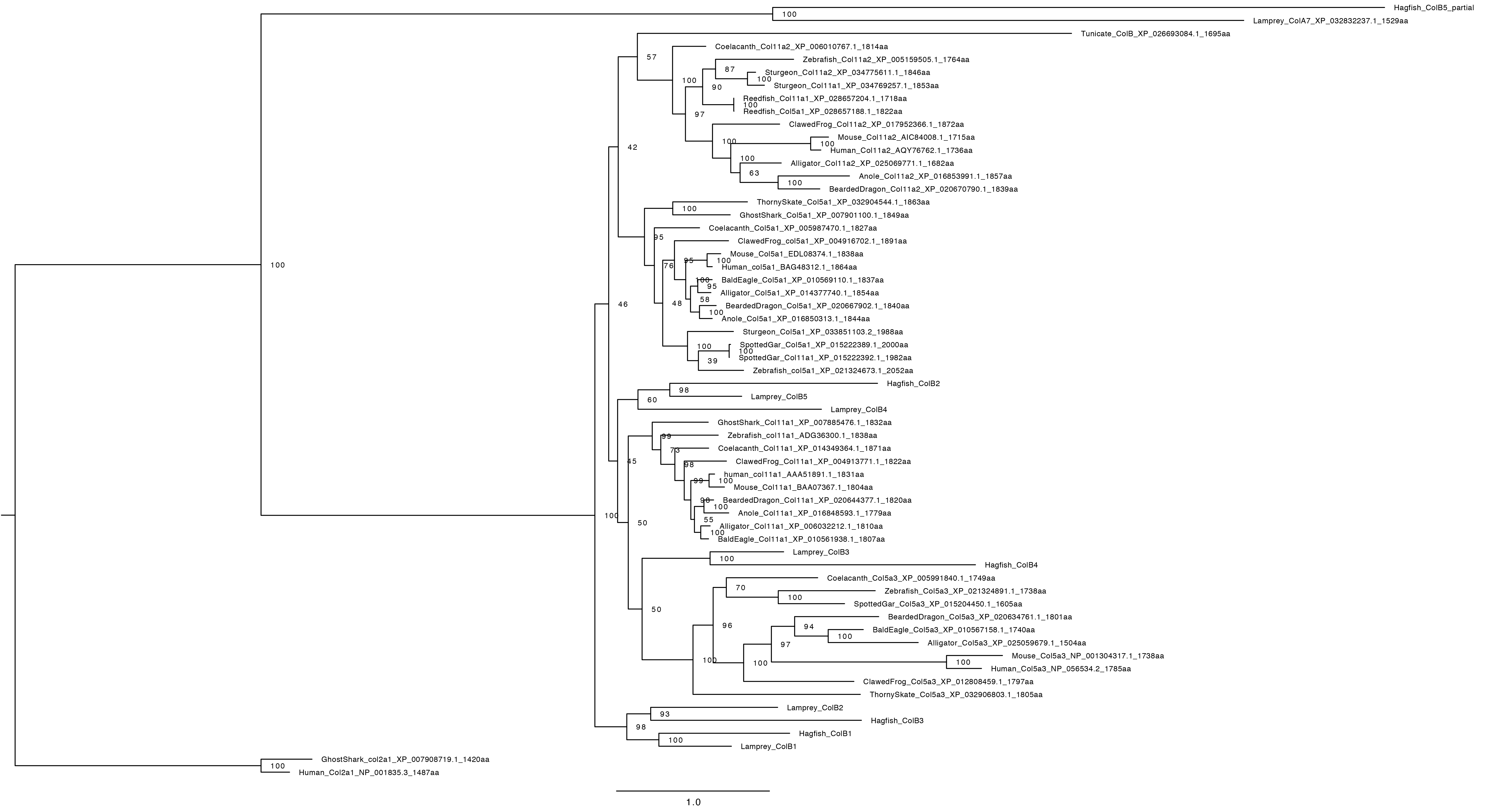

Supplement: Supplementary file 8 [file Image5.jpeg]

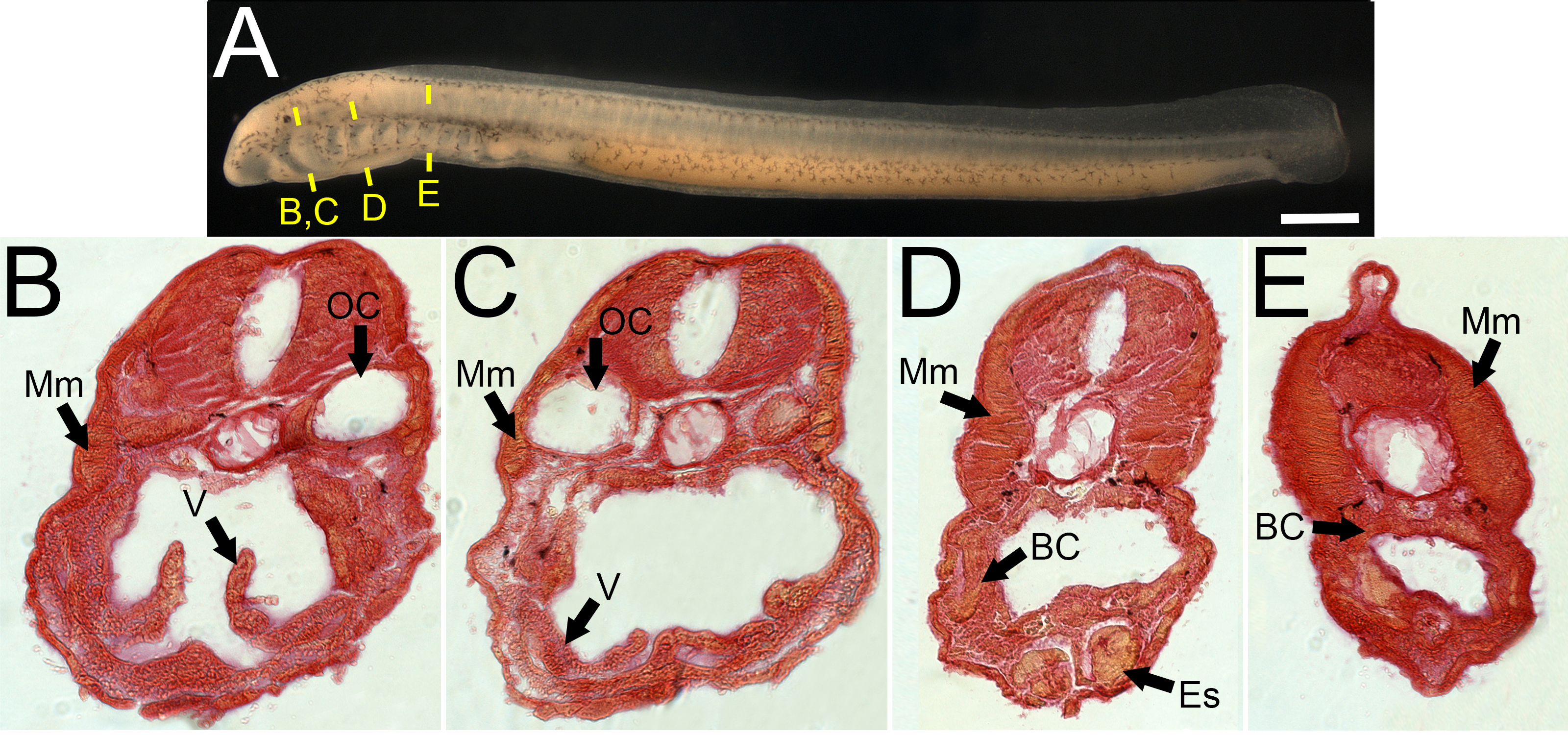

Supplement: Supplementary file 13 [file Image8.jpeg]

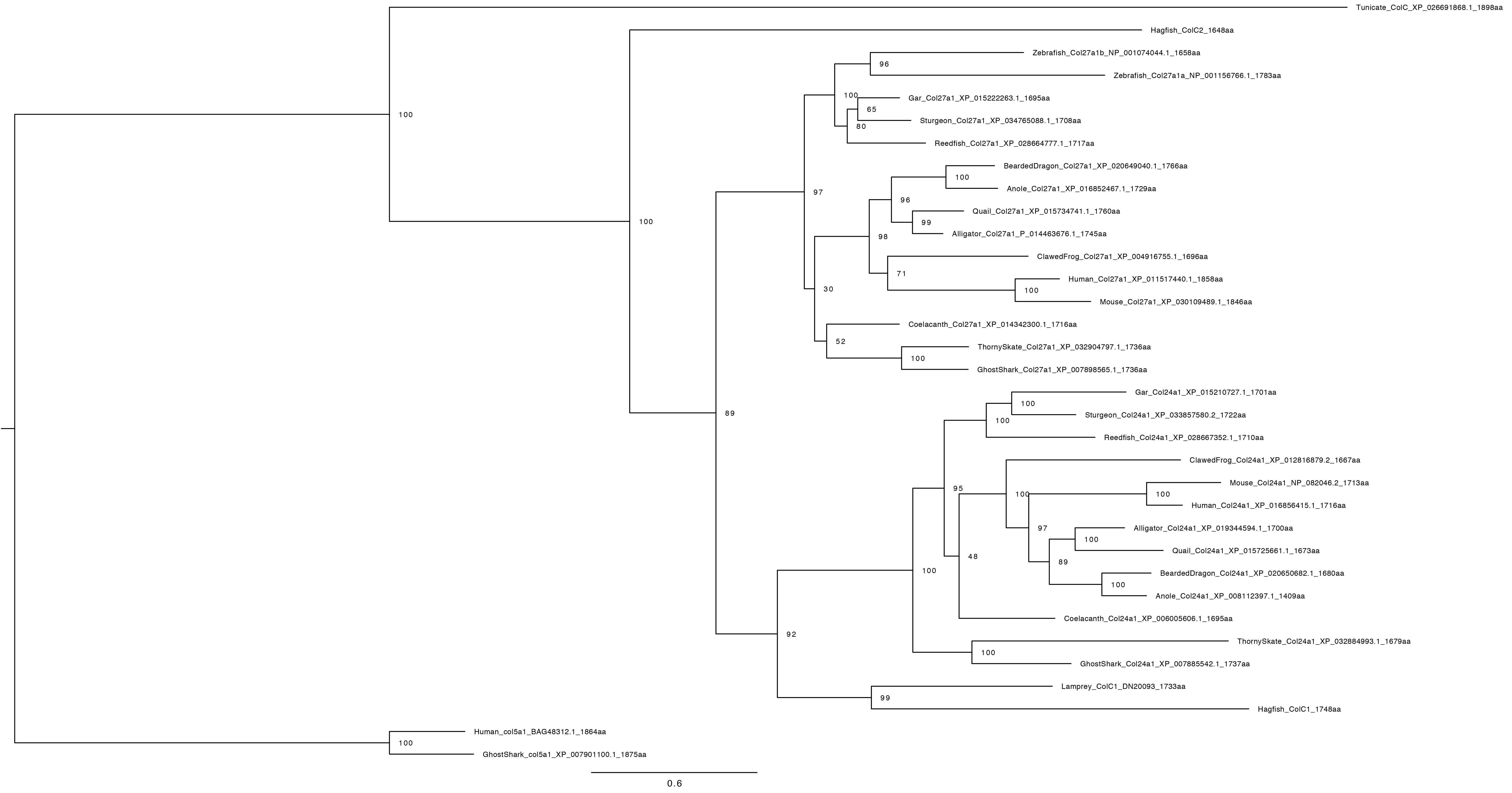

Supplement: Supplementary file 15 [file Image6.jpeg]
