# Supplementary material for: A Comprehensive Analysis of Fibrillar Collagens in Lamprey Suggests a Conserved Role in Vertebrate Musculoskeletal Evolution
Source: Front Cell Dev Biol. 2022 Feb 15;10:809979. doi: 10.3389/fcell.2022.809979 (PMC8887668; doi:10.3389/fcell.2022.809979)
Supplement: Supplementary file 6 [file Table7.docx]

**Table S7.** NCBI accession numbers used for the Clade C phylogenetic analysis in Figure S6.

| **Sequence Name** | **Accession Number** |
| --- | --- |
| Lamprey_ColC1 | OK655910 |
| Hagfish_ColC2 | OK655922 |
| Hagfish_ColC2 | OK655923 |
| Human_Col27a1 | XP_011517440.1 |
| Human_Col24a1 | XP_016856415.1 |
| ClawedFrog_Col27a1 | XP_004916755.1 |
| ClawedFrog_Col24a1 | XP_012816879.2 |
| Zebrafish_Col27a1a | NP_001156766.1 |
| Zebrafish_Col27a1b | NP_001074044.1 |
| Alligator_Col27a1 | XP_014463676.1 |
| Alligator_Col24a1 | XP_019344594.1 |
| ThornySkate_Col27a1 | XP_032904797.1 |
| ThornySkate_Col24a1 | XP_032884993.1 |
| GhostShark_Col27a1 | XP_007898565.1 |
| GhostShark_Col24a1 | XP_007885542.1 |
| Gar_Col27a1 | XP_015222263.1 |
| Gar_Col24a1 | XP_015210727.1 |
| Sturgeon_Col27a1 | XP_034765088.1 |
| Sturgeon_Col24a1 | XP_033857580.2 |
| Reedfish_Col27a1 | XP_028664777.1 |
| Reedfish_Col24a1 | XP_028667352.1 |
| Anole_Col27a1 | XP_016852467.1 |
| Anole_Col24a1 | XP_008112397.1 |
| BeardedDragon_Col27a1 | XP_020649040.1 |
| BeardedDragon_Col24a1 | XP_020650682.1 |
| Coelacanth_Col27a1 | XP_014342300.1 |
| Coelacanth_Col24a1 | XP_006005606.1 |
| Quail_Col27a1 | XP_015734741.1 |
| Quail_Col24a1 | XP_015725661.1 |
| Mouse_Col27a1 | XP_030109489.1 |
| Mouse_Col24a1 | NP_082046.2 |
| Tunicate_ColC | XP_026691868.1 |
| Human_Col5a1 | BAG48312.1 |
| GhostShark_Col5a1 | XP_007901100.1 |
