# Supplementary material for: A Comprehensive Analysis of Fibrillar Collagens in Lamprey Suggests a Conserved Role in Vertebrate Musculoskeletal Evolution
Source: Front Cell Dev Biol. 2022 Feb 15;10:809979. doi: 10.3389/fcell.2022.809979 (PMC8887668; doi:10.3389/fcell.2022.809979)
Supplement: Supplementary file 9 [file Table2.docx]

**Table S2.** NCBI accession numbers used for the Clade B phylogenetic analysis in Figure S2

| **Sequence Name** | **Accession Number** |
| --- | --- |
| Lamprey_ColB1 | OK655905 |
| Lamprey_ColB2 | OK655906 |
| Lamprey_ColB3 | OK655907 |
| Lamprey_ColB4 | OK655908 |
| Lamprey_ColB5 | OK655909 |
| Hagfish_ColB1 | OK655917 |
| Hagfish_ColB2 | OK655918 |
| Hagfish_ColB3 | OK655919 |
| Hagfish_ColB4 | OK655920 |
| Hagfish_ColB5_partial | OK655921 |
| Human_Col11a2 | AQY76762.1 |
| Human_Col5a1 | BAG48312.1 |
| Human_Col5a3 | NP_056534.2_ |
| Human_Col11a1 | AAA51891.1 |
| ClawedFrog_Col11a2 | XP_017952366.1 |
| ClawedFrog_Col5a1 | XP_004916702.1 |
| ClawedFrog_Col5a3 | XP_012808459.1 |
| ClawedFrog_Col11a1 | XP_004913771.1 |
| Zebrafish_Col11a2 | XP_005159505.1 |
| Zebrafish_Col5a1 | XP_021324673.1 |
| Zebrafish_Col5a3 | XP_021324891.1 |
| Zebrafish_Col11a1 | ADG36300.1 |
| BeardedDragon_Col11a2 | XP_020670790.1 |
| BeardedDragon_Col5a1 | XP_020667902.1 |
| BeardedDragon_Col5a3 | XP_020634761.1 |
| BeardedDragon_Col11a1 | XP_020644377.1 |
| Mouse_Col11a2 | AIC84008.1 |
| Mouse_Col5a1 | EDL08374.1 |
| Mouse_Col5a3 | NP_001304317.1 |
| Mouse_Col11a1 | BAA07367.1 |
| SpottedGar_Col5a1 | XP_015222389.1 |
| SpottedGar_Col5a3 | XP_015204450.1 |
| SpottedGar_Col11a1 | XP_015222392.1 |
| Sturgeon_Col11a2 | XP_034775611.1 |
| Sturgeon_Col5a1 | XP_033851103.2 |
| Sturgeon_Col11a1 | XP_034769257.1 |
| BaldEagle_Col5a1 | XP_010569110.1 |
| BaldEagle_Col5a3 | XP_010567158.1 |
| BaldEagle_Col11a1 | XP_010561938.1 |
| GhostShark_Col5a1 | XP_007901100.1 |
| ThornySkate_Col5a3 | XP_032906803.1 |
| GhostShark_Col11a1 | XP_007885476.1 |
| ThornySkate_Col5a1 | XP_032904544.1 |
| Amphioxus_ColB1 | XP_035683323.1 |
| PurpleUrchin_ColB1 | XP_030853889.1 |
| Leech_ColB1 | XP_009016121.1 |
| Human_Col2a1 | NP_001835.3 |
| GhostShark_Col2a1 | XP_007908719.1 |
