# Supplementary material for: A Comprehensive Analysis of Fibrillar Collagens in Lamprey Suggests a Conserved Role in Vertebrate Musculoskeletal Evolution
Source: Front Cell Dev Biol. 2022 Feb 15;10:809979. doi: 10.3389/fcell.2022.809979 (PMC8887668; doi:10.3389/fcell.2022.809979)
Supplement: Supplementary file 11 [file Table3.docx]

**Table S3.** NCBI accession numbers used for the Clade A phylogenetic analysis in Figure 1

| **Sequence Name** | **Accession Number** |
| --- | --- |
| Lamprey_ColA1 | OK655898 |
| Lamprey_ColA2 | OK655899 |
| Lamprey_ColA3 | OK655900 |
| Lamprey_ColA4 | OK655901 |
| Lamprey_ColA5 | OK655902 |
| Lamprey_ColA6 | OK655903 |
| Lamprey_ColA7 | XP_032832237.1 |
| Hagfish_ColA1 | OK655911 |
| Hagfish_ColA2 | OK655912 |
| Hagfish_ColA3 | OK655913 |
| Hagfish_ColA4 | OK655914 |
| Hagfish_ColA5 | OK655915 |
| Hagfish_ColA6 | OK655916 |
| GhostShark_Col1a2 | XP_007907447.1 |
| GhostShark_Col5a2 | XP_007888413.1 |
| GhostShark_Col3a1 | XP_007888321.1 |
| Mouse_Col2a1 | NP_112440.2 |
| Mouse_Col1a1 | NP_031768.2 |
| Mouse_Col1a2 | NP_031769.2 |
| Mouse_Col5a2 | NP_031763.2 |
| Mouse_Col3a1 | NP_034060.2 |
| SpottedGar_Col2a1 | XP_006629302.1 |
| SpottedGar_Col1a1 | XP_006638297.1 |
| SpottedGar_Col1a2 | XP_015213472.1 |
| SpottedGar_Col5a2 | XP_015214430.1 |
| SpottedGar_Col3a1 | XP_015214462.1 |
| Sturgeon_Col2a1 | XP_034769756.1 |
| Sturgeon_Col1a1 | XP_033911740.1 |
| Sturgeon_Col1a2 | RXM37299.1 |
| Sturgeon_Col5a2 | XP_033900774.1 |
| Sturgeon_Col3a1 | XP_033861860.2 |
| ThornySkate_Col2a1 | XP_032871636.1 |
| ThornySkate_Col1a1 | XP_032891216.1 |
| ThornySkate_Col1a2 | XP_032898452.1 |
| ThornySkate_Col5a2 | XP_032879676.1 |
| ThornySkate_Col3a1 | XP_032879677.1 |
| BeardedDragon_Col2a1 | XP_020635061.1 |
| BeardedDragon_Col1a1 | XP_020649626.1 |
| BeardedDragon_Col1a2 | XP_020640818.1 |
| BeardedDragon_Col5a2 | XP_020658514.1 |
| BeardedDragon_Col3a1 | XP_020658565.1 |
| Quail_Col2a1 | XP_015705871.1 |
| Quail_Col1a1 | XP_015741354.1 |
| Quail_Col1a2 | XP_015709029.1 |
| Quail_Col5a2 | XP_015723324.1 |
| Quail_Col3a1 | XP_015723325.1 |
| Alligator_Col2a1 | XP_014465538.1 |
| Alligator_Col1a1 | XP_006277120.1 |
| Alligator_Col1a2 | XP_006258514.1 |
| Alligator_Col5a2 | XP_006258205.2 |
| Alligator_Col3a1 | XP_014449841.1 |
| Coelacanth_Col2a1 | XP_005986344.1 |
| Coelacanth_Col1a1 | XP_005992246.1 |
| Coelacanth_Col1a2 | XP_006011686.1 |
| Coelacanth_Col5a2 | XP_005996582.1 |
| Coelacanth_Col3a1 | XP_005996581.1 |
| Reedfish_Col2a1 | XP_028654356.1 |
| Reedfish_Col1a1 | XP_028674369.1 |
| Reedfish_Col1a2 | XP_028672909.1 |
| Reedfish_Col5a2 | XP_028662025.1 |
| Reedfish_Col3a1 | XP_028661875.1 |
| Anole_Col2a1 | XP_008119414.1 |
| Anole_Col1a1 | XP_003222687.1 |
| Anole_Col1a2 | XP_008110742.2 |
| Anole_Col5a2 | XP_008115789.2 |
| Anole_Col3a1 | XP_008115785.1 |
| Tunicate_ColA1 | XP_026693301.1 |
| Tunicate_ColA2 | XP_018668876.1 |
| Human_Col5a1 | BAG48312.1 |
| GhostShark_Col5a1 | XP_007901100.1 |
